# Free Sterol Content of *Brassica* Microgreens, Microleaves and Baby Leaves: A Quantitative Study by RPLC-APCI-HRMS

**DOI:** 10.3390/molecules31142553

**Published:** 2026-07-22

**Authors:** Valeria Cinquepalmi, Ilario Losito, Andrea Castellaneta, Beniamino Leoni, Massimiliano Renna, Onofrio Davide Palmitessa, Pietro Santamaria, Cosima Damiana Calvano, Tommaso R. I. Cataldi

**Affiliations:** 1 Dipartimento di Chimica, Università degli Studi di Bari Aldo Moro, Via Orabona 4, 70126 Bari, Italy; valeria.cinquepalmi@uniba.it (V.C.); andrea.castellaneta@uniba.it (A.C.); cosimadamiana.calvano@uniba.it (C.D.C.); tommaso.cataldi@uniba.it (T.R.I.C.); 2 Centro Interdipartimentale SMART, Università degli Studi di Bari Aldo Moro, Via Orabona 4, 70126 Bari, Italy; pietro.santamaria@uniba.it; 3 Dipartimento di Scienze del Suolo, della Pianta e degli Alimenti, Università degli Studi di Bari Aldo Moro, Via G. Amendola 165/a, 70126 Bari, Italy; beniamino.leoni@uniba.it (B.L.); massimiliano.renna@uniba.it (M.R.); onofrio.palmitessa@uniba.it (O.D.P.)

**Keywords:** free sterols, kale, broccoli raab, microgreens, microleaves, baby leaves, atmospheric pressure chemical ionization high-resolution mass spectrometry

## Abstract

In recent years, innovative edible vegetal products, obtained by harvesting in the early stages of plant development, have emerged for their potential as sources of nutraceuticals. Phytosterols, which play important structural and regulatory roles in plant growth and membrane remodeling, are included among vegetable-related nutraceuticals due to their beneficial effects on human health. For this reason, the present study was focused on the use of RPLC-APCI-HRMS to assess, for the first time, the free sterol (FS) profiles of two edible *Brassicaceae* plants, namely kale (*Brassica oleracea* var. *acephala*) and broccoli raab (*Brassica rapa* subsp. *sylvestris* var. *esculenta*), harvested at the stages of microgreens, microleaves and baby leaves. A comparison with the respective adult forms, conventionally used as edible products upon cooking, was also performed. β-sitosterol was the prevailing FS in all products (>75%), followed by campesterol (8–15%) and isofucosterol (<3%). Total FS concentrations ranged between 13 and 23 mg/100 g fresh weight (FW) for juvenile products of the two plants under study, with the respective highest values being comparable with those found in raw or cooked mature forms (24–26 mg/100 g FW). Interestingly, a more diversified FS profile was observed for juvenile products and an increase in major FS content was observed for microleaves of the two plants cultivated during the winter cycle (January–March), compared to those cultivated in the spring cycle (March–May). These results support the hypothesis that vegetables harvested at early developmental stages may represent promising ready-to-eat alternatives to *Brassica* plant adult forms, as well as sources of beneficial phytosterols.

## 1. Introduction

In recent years, edible plant products harvested at early developmental stages have gained increasing attention in the agri-food sector, not only because of their short cultivation cycle and reduced environmental footprint, but also because juvenile tissues often display higher concentrations of health-related metabolites with respect to those of mature vegetables [1]. Among these products, microgreens, i.e., tender seedlings harvested 7 to 21 days after sowing, once the cotyledons are expanded and the first true leaves have emerged, have been the major focus of research [2,3,4,5,6,7,8,9]. They are typically cultivated in protected or soilless systems that reduce water use and agrochemical inputs, and are therefore regarded as promising components of sustainable horticultural production [1,10,11,12]. Production-oriented studies have also examined nutrient solution management, use of low-potassium supply, agronomic biofortification, and the effects of irradiation by LED lights on microgreen composition and quality [13,14,15,16,17,18,19,20,21,22,23,24]. Baby leaves, defined by European Regulation 752/2014 as young leaves and petioles harvested up to the stage of the eighth true leaf [1], have also been investigated as nutritionally valuable alternatives to mature vegetables [25,26,27,28,29]. By contrast, the intermediate growth phase, here termed microleaf, corresponding to seedlings with fully expanded first true leaves, has received much less attention, despite its potential relevance for both plant physiology and product quality. This stage represents a critical transition period in plant development, during which seedlings shift from heterotrophic growth based on seed reserves to autotrophic growth supported by photosynthesis. It is also characterized by rapid leaf expansion and active cellular membrane biogenesis, processes in which sterols, which represent the target molecules of the present investigation, play essential structural and regulatory roles.

Based on this background, the present study was designed to assess, for the first time, the profile and content of an important class of nutraceuticals, i.e., phytosterols, in microgreens, microleaves and baby leaves of two plants of the *Brassicaceae* family, namely kale (*Brassica oleracea* L. var. *acephala*) and broccoli raab (*Brassica rapa* L. subsp. *sylvestris* L. Janch. var. *esculenta* Hort.), whose adult forms are very popular among edible plants consumed in Southern Italy and are well known for their richness in bioactive constituents such as polyphenols and glucosinolates [30,31,32,33,34,35,36,37,38]. In this case, attention was directed to phytosterols (PSs), not only because of their recognized benefits for human health, including LDL-cholesterol-lowering effects and other health-promoting properties [39,40,41], but also because they are directly involved in plant growth processes that are especially dynamic during the transition from seedlings to mature edible tissues.

**Figure 1:**
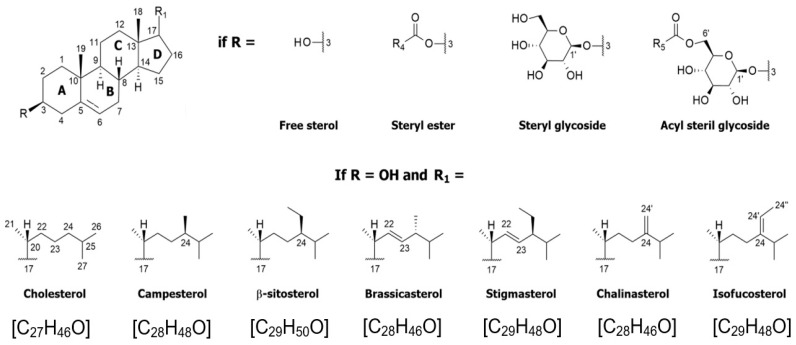
(**Top Left**) General trans-planar tetracyclic structure of Δ^5^-sterols, numbered according to IUPAC nomenclature. (**Top Right**) General structures of the R group linked to C3, determining sterol classification as free sterols (FSs), steryl esters (SEs), steryl glycosides (SGs) and acylated steryl glycosides (ASGs). (**Bottom**) Side chains, indicated as R_1_, of the most relevant plant FSs and of cholesterol, considered in the present study along with the Δ^7^ analog of isofucosterol, i.e., Δ^7^ avenasterol.

PSs are key structural components of plant cell membranes, where they regulate permeability, fluidity and lipid bilayer organization, while also participating in signaling, membrane trafficking and plasmodesmata function [41,42,43,44,45,46]. Significantly for the present study, sterol metabolism is tightly connected with plant growth and development because sterols influence cell division, elongation and differentiation [43,46,47], and their relative abundance is known to respond to developmental cues and abiotic conditions, including temperature [46,48]. As evidenced in Figure 1, PSs are present in plants mainly as free sterols (FSs), although steryl esters (SEs), steryl glycosides (SGs) and acylated steryl glycosides (ASGs) can also be found in vegetal tissues [41,43,49,50] and are increasingly recognized as functionally relevant metabolites, yet their analytical characterization requires careful hydrolysis strategies for conversion into the corresponding free forms [51,52]. Among PSs differing for the extent of methylation at C4 of the common cyclopenta[α]phenanthrene tetracyclic structure, i.e., desmethyl-, 4α-methyl-, and 4,4-dimethylsterols [41,50,53], the latter are less abundant in plant tissues, since they serve mainly as precursors of demethylated sterols [54]. Considering the position of C=C bond(s) on the steroidal B-ring, FSs are additionally classified as Δ^5^-, Δ^7^-, Δ^5,7^-, or Δ^8^-sterols, whereas stanols are fully saturated compounds [41,44,50]. Δ_5_-sterols, on which Figure 1 is focused (note that the IUPAC numbering of sterol carbon atoms [55] is adopted in the figure), include β-sitosterol and campesterol, the most abundant FSs in plants, possessing a fully saturated side chain branched at C24 [39,43,50] through enzymatic ethylation or methylation [43,46,56,57]. Other common plant Δ^5^-sterols, such as brassicasterol and stigmasterol, include a C=C double bond between C22 and C23 [41,43], whereas chalinasterol and isofucosterol are their respective positional isomers (Figure 1). The Δ^7^ isomer of isofucosterol, known as Δ^7^-avenasterol, is also found in plants.

Several studies have investigated FSs in mature *Brassica* vegetables [58,59,60,61,62,63,64,65]; however, information on juvenile *Brassica* products is still scarce. To date, only one study has examined phytosterols in *Brassica* microgreens, using GC-MS/MS [66], while no work has systematically compared microgreens, microleaves, baby leaves and mature vegetables across developmental stages. This systematic study was thus performed for the first time in the present work, starting from an RPLC-APCI-HRMS method previously developed in our laboratory, enabling the detailed characterization of major plant sterols and the interpretation of their APCI-HRMS/MS fragmentation behavior [67]. We hypothesized that the free sterol composition undergoes systematic changes during early *Brassica* development owing to the intense membrane remodeling associated with seedling growth. To test this hypothesis, the RPLC-APCI-HRMS method was first validated for quantitative application and then applied to quantify sterols in microgreens, microleaves, baby leaves and mature tissues. In this way, the study was also meant to address the question of whether juvenile *Brassica* products can realistically be considered alternatives to the corresponding adult forms as sources of free sterols. Moreover, the eventual effect of cultivation season was also tested in the case of microgreens and microleaves.

## 2. Results and Discussion

### 2.1. Identification of Sterols by RPLC-APCI(+)-HRMS and HRMS/MS Analysis

**Figure 2:**
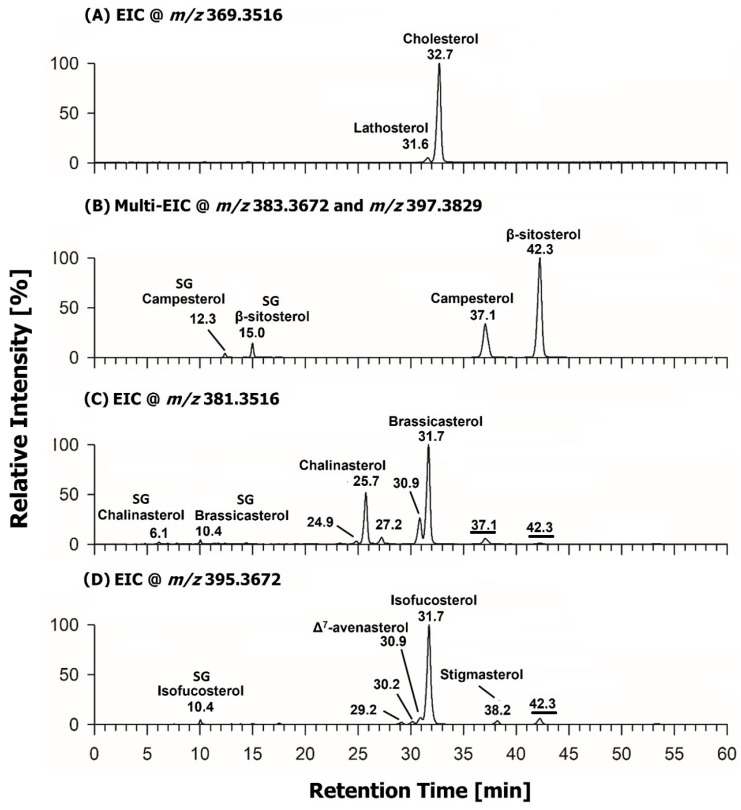
Extracted ion current (EIC) chromatograms referring to monoisotopic *m*/*z* values for [M + H − H_2_O]^+^ ions of sterols identified in a kale microgreen extract after RPLC-APCI-HRMS analysis: (**A**) 369.3516, lathosterol and cholesterol; (**B**) 383.3672 and 397.3829, campesterol and β-sitosterol, respectively; (**C**) 381.3516, chalinasterol and brassicasterol; (**D**) 395.3672, Δ^7^-avenasterol, isofucosterol (Δ^5^-avenasterol) and stigmasterol. Labeled peaks correspond to FS identified using authentic standards; peaks at earlier retention times correspond to the related steryl glycosides (SG). Underlined retention times (campesterol, 37.1 min; β-sitosterol, 42.3 min) indicate peaks arising from in-source fragmentation of the respective major sterol molecular ions. Non-underlined retention times correspond to minor sterols tentatively identified using chromatographic and tandem mass spectrometric data (see text for details).

In the first stage of the present study, a detailed assessment of FS profiles in the vegetal products of interest was undertaken, also considering minor compounds of the same class, that could contribute to stage-dependent differences in FS composition. The RPLC-APCI(+)-HRMS method adopted for FS analysis provided a comprehensive profile of these compounds for all vegetal products considered in this study. As an example, single or multiple extracted ion current (EIC) chromatograms corresponding to the monoisotopic *m*/*z* values of FS [M + H − H_2_O]^+^ ions detected in a kale microgreen extract are reported in Figure 2, with labels indicating either free sterols, recognized through comparison of retention times and accurate *m*/*z* ratios of the respective ions with those of standards, or the respective steryl glycosides (SGs). Notably, [M + H − H_2_O]^+^ ions prevailed remarkably with respect to [M + H]^+^ ones for all the sterols under study in the adopted APCI conditions.

In this case, lathosterol was successfully separated from cholesterol (Figure 2A), in accordance with the previously demonstrated ability of the developed RPLC gradient to resolve Δ^7^ from Δ^5^ sterols [67,68]. Although more characteristic of animal matrices, the presence of lathosterol and cholesterol is not unexpected, as these sterols have been previously detected in mature *Brassica* vegetables as well as in kale and broccoli raab microgreens, with cholesterol typically representing about 1% of the total sterol content in plants [44,64,66,69]. Besides those of campesterol and β-sitosterol, two further peaks were observed, at 12.3 and 15.0 min, in the multi-EIC trace corresponding to [M + H − H_2_O]^+^ ions with exact *m*/*z* values 383.3672 and 397.3829 (Figure 2B). They could be related to the corresponding SGs, that were co-extracted with their free counterparts but were eluted earlier due to the presence of the polar sugar moiety, which reduces the interaction with the C18 stationary phase. The SG protonated forms underwent the neutral loss of the sugar moiety during the APCI process, thus yielding the same ions observed for free sterols, as confirmed also by their HCD-MS/MS spectra. Chalinasterol and brassicasterol were readily recognized in the EIC chromatogram obtained for *m*/*z* 381.3516, while peaks eluting at 6.1 and 10.4 min corresponded to the respective SGs (Figure 2C). Additional weak chromatographic peaks detected after 37.1 and 42.3 min in the same EIC chromatogram were found to correspond to product ions resulting from the [M + H − H_2_O]^+^ ions of campesterol and β-sitosterol upon APCI-induced neutral losses of H_2_ and CH_4_, respectively. Finally, peaks corresponding to isomeric Δ^7^-avenasterol and isofucosterol (also known as Δ^5^-avenasterol), as well as the very weak peak related to stigmasterol, were readily recognized in the EIC trace obtained for *m*/*z* 395.3672 (Figure 2D). The peak detected at 10.4 min in this chromatogram corresponded to the SG of isofucosterol, as demonstrated by the HCD-MS/MS spectrum of the corresponding standard. Notably, a perfect alignment was observed for peaks related to brassicasterol and isofucosterol SGs, by analogy with their free forms. The last peak in plot D, eluted after 42.3 min, was related to the product ion resulting from the [M + H − H_2_O]^+^ ion of β-sitosterol upon H_2_ loss in the APCI source.

The detection of additional weak peaks in the EIC chromatograms corresponding to *m*/*z* values 381.3516 (24.9, 27.2 and 30.9 min) and 395.3672 (29.2 and 30.2 min) suggested the presence of additional sterols. The identification of these compounds was attempted by integrating chromatographic and mass spectrometric data with information available on sterols in the dedicated section of the LipidMaps database (www.lipidmaps.org). In fact, our previous studies on phytosterols and animal/fungal sterols demonstrated the existence of several diagnostic correlations between relative intensities of product ions detected in HCD-MS/MS spectra and important structural features, including the number and position of double bonds on the B ring and the characteristics (unsaturation, branching) of the side chain [67,68]. APCI-HCD-MS/MS spectra were therefore carefully averaged under peaks detected in the EIC chromatograms of Figure 2 that could not be identified through comparison with major sterol standards and are reported in Figure S1 of the Supplementary Material. They were subsequently employed, along with chromatographic data and a comparison with the literature [69,70,71,72,73], to obtain structural information on minor sterols, as explained in detail in Section S1 and Figures S2 and S3 of the Supplementary Materials.

As a result, the following putative assignments were obtained for peaks detected in the EIC chromatogram at *m*/*z* 381.3516 (Figure 2C): (24.9 min) episterol (24-methylene-cholest-7-en-3β-ol); (27.2 min) 24-methylcholesta-5,23E-dien-3β-ol; (30.9 min) 24-epibrassicasterol (also known as campesta-5,22E-dien-3β-ol or crinosterol). These assignments were obtained for minor peaks detected in the EIC chromatogram at *m*/*z* 395.3672 (Figure 2D): (29.2 min) 14-demethyl-lanosterol (also known as 4,4-dimethylzymosterol or 4,4-dimethyl-5α-cholesta-8,24-dien-3β-ol); (30.2 min) Δ^5,24^-stigmastadienol.

As a general comment on the sterols identified in kale microgreens, it is worth noting that chalinasterol was previously detected in vegetal matrices only in *Cucurbitaceae* plants [74,75,76]. To the best of our knowledge, episterol and 24-epibrassicasterol have never been identified in vegetables, whereas they were found in fungi and algae respectively [77,78]. Similarly, 14-demethyl-lanosterol is a free sterol typically found in fungi [79]. Δ^5,24^-stigmastadienol has been identified so far only in plants belonging to *Graminaceae*, *Fabaceae*, and *Solanaceae* families [80,81,82], but never in *Brassicaceae*. Therefore, the RPLC-APCI-HRMS/MS approach adopted during this study enabled us to extend the knowledge on the sterol profile of these plants. As discussed in the following sections, similar profiles were observed among the different products of kale and broccoli raab on a qualitative level. However, interesting differences were observed from a quantitative point of view.

### 2.2. Quantification of Sterols: Method Development and Validation

As a first step towards the quantification of sterols in vegetal products, six-level external calibration was performed on the set of sterol standards considered in the present study. The analytical response was obtained from the EIC peak areas referring to [M + H − H_2_O]^+^ ions, normalized by the peak area of the [M + H − H_2_O]^+^ ion cholesterol-d_6_, added to each sample as the internal standard (IS) at a 10 μg/mL final concentration (see Section 3). Notably, in this case cholesterol-d_6_ was added with the specific purpose of compensating for eventual instrumental response fluctuations, which are not uncommon in APCI-MS instruments. In particular, EIC peak areas obtained for each of the target standard sterols were systematically normalized by the EIC peak areas obtained for cholesterol-d_6_, under the assumption that any instrumental response fluctuation would influence the respective responses similarly. As evidenced in Table S1 (Supplementary Materials), response linearity in the explored concentration ranges was consistently excellent and the limits of detection (LOD) and quantification (LOQ), calculated as 3 and 10 times the s/b ratio, respectively, where s is the standard deviation of the intercept and b is the slope of the calibration line [83], ranged, respectively, between 0.03 and 2 μg/mL (LOD) and between 0.11 and 7 μg/mL (LOQ).

The matrix effect was subsequently evaluated by the standard addition method, since no blank matrix was available. Each standard sterol was added to extracts of microgreens, microleaves, baby leaves and mature forms of both kale and broccoli raab to reach the third and the sixth concentration level used for calibration (see Section 3). Three spiking replicates were considered in each case, and the matrix effect was calculated as the ratio between the IS-normalized EIC peak area obtained for each of the two levels spiked in the matrix, subtracted of the IS-normalized EIC peak area found in the unspiked matrix (thus reflecting the sterol content originally present in the matrix), and the IS-normalized EIC peak area obtained for the same concentration in solvent. Values between 90 and 110% were obtained for all sterols, indicating negligible matrix effects in all the matrices of interest. Consequently, external calibrations curves could be reliably employed for sterol quantifications in real samples. For the five sterols described in the previous section for which standards were unavailable, quantification was based on calibration lines of structurally similar sterols eluting at comparable retention times. Indeed, as discussed in our previous papers on the APCI-MS and MS/MS analysis of sterols, the similarity between sterol molecular structures is expected to lead to similar efficiencies for the generation of the corresponding [M + H − H_2_O]^+^ ions during the APCI process, being ionization efficiencies related to the probability of formation of specific sterol carbocations following protonation and loss of water, in turn influenced by molecular structure [67,68]. Additionally, the initial transfer of H^+^ to the neutral sterol molecule during the APCI process is clearly expected to be influenced by the composition of the mobile phase; thus, given a certain level of structural similarity, a similar ionization efficiency can be hypothesized if two sterols are also eluted at similar retention times from the C18 column.

Based on these considerations, episterol and 24-methylcholesta-5,23-dien-3β-ol were quantified using the chalinasterol calibration line, since the former is the Δ^7^ equivalent of chalinasterol and the latter shares with chalinasterol the C=C bond between C5 and C6 and the occurrence of a C=C bond along the side chain. Brassicasterol calibration was used also for its epimer 24-epi-brassicasterol, whereas 14-demethyl-lanosterol and Δ^5,24^-stigmastadienol were quantified using the calibration line of isofucosterol, since both those sterols share with isofucosterol the presence of a C=C bond close to the end of their side chain.

The recovery of sterols from the vegetal matrices was estimated in triplicate using kale microgreens as a model matrix. Three successive extraction steps were performed in each case, with the resulting supernatants collected and evaporated under nitrogen flow, and the resulting residues dissolved into 500 μL of a CH_3_OH/CHCl_3_ solution (2:1 *v*/*v*). Before RPLC-APCI(+)-HRMS analysis, the solutions were diluted 1:5 (*v*/*v*) with acetonitrile and cholesterol-d_6_ was added as the internal standard (IS) at a 10 μg/mL final concentration. The IS-normalized EIC peak area of each sterol for each extraction step could thus be correlated to the extracted amount and expressed as the percentage of the total peak area obtained after three extraction steps. Average percentual recovery values obtained after each extraction step, reported in Figure S4 (Supplementary Materials), showed that at least 95% of the sterol amount obtained after three consecutive extractions could be extracted already after the first two steps. Moreover, the variabilities of recoveries did not exceed 7% for the first extraction and 5% for the second one, confirming the reproducibility and reliability of the adopted extraction protocol. Accordingly, the pooled first and second extracts were used for real-sample analysis, as described in detail in Section 3.

As a final step of method validation, response variabilities related either to between-day instrumental fluctuations or to sampling of freeze-dried vegetal samples combined with extraction yield reproducibility were assessed as described in detail in Section S2 of the Supplementary Materials. In the former case, RSD values were found to be lower than 6% (see Table S2 in the Supplementary Materials), thus confirming the efficacy of response normalization based on the isotopically labeled internal standard and the stability of sterols in extracts over a typical analytical timescale (5 days). In the latter case, RSD was generally lower than 15%, being close to 20–21% only for brassicasterol and 24-epibrassicasterol (see Table S2). The relatively higher values found for the last two sterols are likely related to the intrinsic variability of their amounts between different individual plants, which could not be compensated even by pooling the lyophilized material obtained from several plants grown in a specific sub-section of the greenhouse, as described in detail in Section 3. As evidenced in the next section, brassicasterol and 24-epibrassicasterol were critical sterols, since their concentrations became so low, already at the level of microleaves, to become undetectable in products different from microgreens among those considered for the present study. It is thus likely that a more pronounced variability occurred for the contents of these sterols in different individual plants already at the level of microgreens.

### 2.3. RPLC-APCI-HRMS-Based Quantification of Free Sterols in Juvenile Products and Adult Forms of Kale and Broccoli Raab

**Figure 3:**
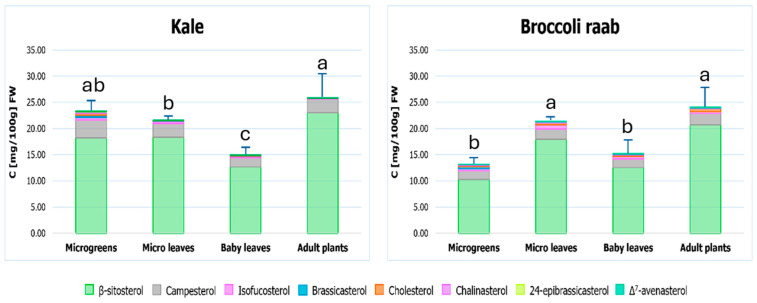
Stacked-column graphs illustrating the average total contents of free sterols (FS) found in microgreens, microleaves, baby leaves and adult forms of kale and broccoli raab (*n* = 3). Contents are expressed as mg/100 g fresh weight (FW). Error bars represent the 95% confidence intervals for the total sterol concentrations reported for each product. Letters indicate the outcome of one-way ANOVA with Tukey’s HSD post hoc test (*p* = 0.05).

The first step of the quantitative assessment of FSs involved microgreens, microleaves, baby leaves and mature forms of both kale and broccoli raab grown in the winter season (January–March), which represents the typical cultivation period for the mature vegetables. This comparison was designed to test the hypothesis that juvenile products may already accumulate nutritionally relevant FS amounts and to verify if the developmental stage affects not only their total content, but also their profile. A single aliquot of the pooled freeze-dried material obtained from microgreens/microleaves/baby leaves, according to the case, harvested in each of the three specific sub-sections of the greenhouse inside which they were cultivated (see Section 3 for details), was subjected to sterol extraction and the extract was subjected to RPLC-APCI-HRMS analysis. Each extract was analyzed once, considering the excellent reproducibility of the analytical response for the same extract discussed before. As explained in the previous section, quantification was performed using external calibration and average concentrations were expressed as mg per 100 g of fresh weight (FW), considering the water loss occurring during freeze-drying of a specific quantity of fresh product. The resulting data, expressed as 95% confidence intervals centered on means (*n* = 3), are reported in Table 1, in which the outcome of a one-way ANOVA with a post hoc HSD Tukey test (*p* = 0.05) for the comparison between multiple means, referring to the different growth stages of the same plant, is reported using superscript. Stacked column graphs were generated from quantitative data reported in Table 1 to enable an easier comparison of the average total sterol contents of the four product types for each *Brassica* plant and are reported in Figure 3, in which error bars represent the half-width of 95% confidence intervals (*n* = 3) and letters indicate the outcome of one-way ANOVA with post hoc HSD Tukey test (*p* = 0.05).

β-sitosterol clearly exhibited the highest content in all products examined in this work, accounting for 80–90% of the total sterol contents, regardless of the developmental stage. Its prevalence, and the role of campesterol as the second most concentrated sterol (again, in all developmental stages) is in accordance with the literature data on plant sterols, including those reported for *Brassicaceae* plants [39,43,50,59,61,63,64,66]. Conversely, stigmasterol always exhibited concentrations even lower than LOD values; therefore, it is not included in Table 1. This outcome was surprising, since stigmasterol is generally considered the third most abundant free sterol in plants [39,43,50]. However, the same result was recently obtained for adult forms of both kale and broccoli raab after GC-MS analysis [66]. Moreover, low stigmasterol amounts were also reported in the literature for several cruciferous vegetables [59,61,63,64,65]. Isofucosterol ranked as the third most concentrated sterol, independently on the developmental stage considered, whereas its positional isomer, Δ^7^-avenasterol, was present at much lower concentrations (see Table 1). Not surprisingly, cholesterol and chalinasterol, typically considered animal sterols, exhibited low concentrations, becoming lower than LOD values in the case of mature kale. Lathosterol was also detected but not quantifiable in any product examined, like most of the minor sterols putatively identified using chromatographic and MS/MS information (see Section 2.1). The only exception among the latter was 24-epibrassicasterol, which, like its major epimer, brassicasterol, could be quantified only in microgreens of both plants, thus indicating a sharp decrease in their abundance during plant growth. Remarkably, small amounts of brassicasterol have generally been reported for mature cruciferous plants [63,64,66]. Data reported in Table 1 also reveal a generally higher variability in the sterol content of mature kale and broccoli raab. The higher variability observed for mature kale and broccoli raab may reflect a greater variability in cultivation conditions across the field, potentially able to determine slight changes in individual sterol contents, but also a more pronounced heterogeneity across different parts of the adult plant [48,84,85]. More importantly, however, quantitative data support the view that FS accumulation has some dependence on the growth stage in the two plants under study.

Based on current knowledge of sterol biosynthetic pathways [43,68,86], the data described so far suggest that a certain degree of sterol profile remodeling accompanies *Brassica* plant development. In particular, the occurrence of brassicasterol and 24-epibrassicasterol only in microgreens, together with the progressive predominance of β-sitosterol in mature plants, is consistent with a developmental regulation of late sterol biosynthetic steps, including the progressive inhibition or the under-expression of enzymes involved in C22 desaturation (which converts β-sitosterol into stigmasterol and campesterol into brassicasterol and 24-epibrassicasterol). Although the present results do not prove a specific enzymatic mechanism, they support the broader interpretation that sterol metabolism is actively reshaped during the transition from seedlings to mature edible plants.

**Figure 4:**
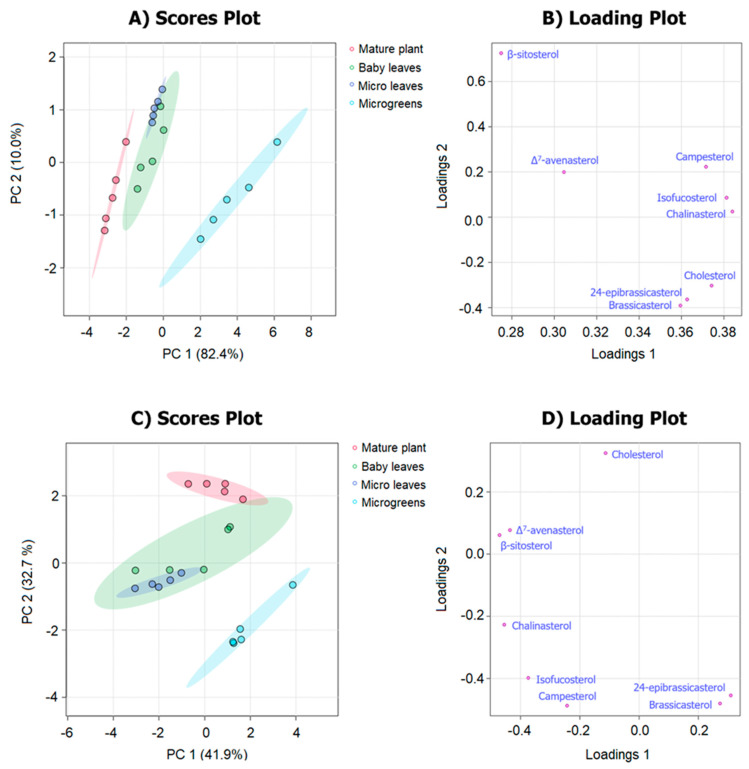
Scores and loading plots obtained for the first two principal components (PC1 and PC2) following PCA of sterol quantitative data for kale (plots (**A**,**B**)) and broccoli raab (plots (**C**,**D**)) microgreens, microleaves, baby leaves and adult plants.

To emphasize differences in terms of sterol profile observed among different products of the same plant, FS quantitative data were used, after autoscaling, as input variables for principal component Analysis (PCA). The resulting scores and loading plots are shown in Figure 4, in which 95% confidence regions for the four product types are also depicted. As a result, kale microgreens and mature form samples were well separated in the score plot, with a higher relevance of Δ^7^-avenasterol and β-sitosterol in the former ones and of brassicasterol and 24-epibrassicasterol in the latter. Microleaf and baby leaf samples occupied intermediate positions and were co-clustered in the PCA score plot due to the greater similarity in terms of sterol profile. Mature forms were clearly distinguished from microgreens in the case of broccoli raab as well, with the higher incidence of brassicasterol and 24-epibrassicasterol being confirmed in microgreens. Taken together, the PCA results reinforce the idea that developmental stage leaves a compositional imprint on FS profiles. The higher incidence of brassicasterol and 24-epibrassicasterol in microgreens of both kale and broccoli raab may reflect a greater requirement for membrane fluidity (enhanced by their trans C=C bond at C22, which weakens van der Waals interactions with membrane lipid hydrocarbon chains) in rapidly growing tissues [41]. On the other hand, the higher prevalence of β-sitosterol in mature forms is consistent with its recognized role in maintaining cell rigidity and membrane integrity [41,43,47]. Notably, the higher cholesterol incidence emphasized by PCA in mature broccoli raab may also deserve further attention in light of the involvement of this sterol in abiotic stress responses and as a precursor of other bioactive metabolites [48,49,50].

To assess whether the developmental effect described above was robust to changes in cultivation conditions, the sterol content was also evaluated for microgreens and microleaves of kale and broccoli raab cultivated and harvested during a spring cycle (March–May).

The resulting average concentrations, expressed as mg per 100 g FW and accompanied by the respective 95% confidence intervals (*n* = 3), are reported in Figure S5 (Supplementary Materials) and directly compared with those obtained for the same products during the winter cycle. Winter-grown microleaves showed significantly higher concentrations of β-sitosterol and campesterol than the corresponding spring-grown products for both plant species. Isofucosterol, cholesterol, chalinasterol and Δ^7^-avenasterol were also statistically more abundant in broccoli raab microleaves cultivated in winter, compared to the respective spring counterparts. The only notable exception to these trends concerning microleaves was the statistically lower concentration of Δ^7^-avenasterol in kale microleaves cultivated in spring (see Figure S5).

Notably, microgreens and microleaves were cultivated without applying artificial heating to the greenhouse; thus, an increasing trend in temperature certainly occurred from the winter to the spring cycle, as clearly evidenced in Figure S6, which reports average, maximum and minimum daily temperature values recorded inside the greenhouse during the two cultivation cycles. The average daily temperature generally ranged between 5 and 10 °C during the winter cycle and between 10 and 20 °C during the spring one.

So far, several studies have been dedicated to assessing the effect of temperature on the accumulation of sterols in plants and an increase in the sterol content has been generally observed upon exposure to low temperatures and interpreted with the hypothesis that colder conditions stimulate sterol accumulation as part of membrane acclimation responses [46,48]. In particular, β-sitosterol and campesterol, i.e., sterols with saturated side chains alkylated on C24, were found to be more concentrated in *Triticum aestivum* exposed to a 4 °C temperature for increasing times [46]. The higher content in these two major sterols observed in our case for both kale and broccoli raab microleaves cultivated during the winter cycle is consistent with those observations. Moreover, our data suggest that the different sterol accumulation between the winter and the spring cycle is related to the developmental stage, since it was generally not observed for microgreens (see Figure S5).

### 2.4. Quantification of Free Sterols in Adult Plants of Kale and Broccoli Raab Subjected to Steaming or Boiling in Water

**Figure 5:**
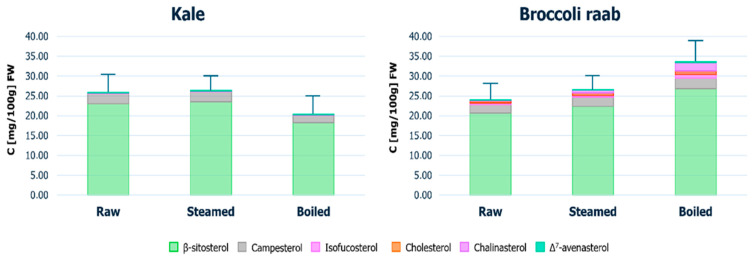
Stacked-column graphs showing free sterol contents in raw, steamed and boiled mature forms of kale and broccoli raab. Concentrations are expressed as mg per 100 g fresh weight (FW) and represent mean values obtained from five samples for raw forms (same data already shown in Figure 3) and from three samples for each cooked vegetable. Error bars indicate the 95% confidence intervals for the total sterol concentrations reported for each product. Note that, in the case of cooked vegetables, 100 g FW are referring to the vegetable after cooking.

As mentioned earlier, mature forms of kale and broccoli raab need to be cooked before consumption, which raises concerns that part of the sterol content present in raw tissues might be lost through thermal degradation. To evaluate the extent of this effect, mature plants of both kale and broccoli raab were subjected to two common cooking procedures, i.e., steaming and boiling in water, with three replicates considered for each condition (see details in Section S4 in the Supplementary Materials). After cooking, samples were freeze-dried prior to sterol extraction and RPLC-APCI-HRMS analysis, following the same analytical workflow adopted for raw plants. Stacked-column plots of the resulting concentrations for single sterols, expressed as mg per 100 g FW (in this case referring to the weight of cooked samples before freeze-drying), along with 95% confidence intervals for total sterol concentrations, are reported in Figure 5, where they are also compared with data referring to raw mature forms already shown before. Concentrations found for single sterols detected in cooked samples are also reported in tabular form in Table S3 (Supplementary Materials). Data on brassicasterol and its epimer were excluded, as their concentrations consistently fell below the LOD in cooked samples, in agreement with observations made on raw forms.

As shown in Figure 5 and Table S3, the two cooking methods generally produced comparable results in terms of final total sterol concentrations in the two plants; in particular, the decrease or increase in total content observed, respectively, for boiled kale and broccoli raab were not great enough to be statistically significant (Figure 5). As for single sterols, Table 1 and Table S3 indicate that statistically comparable amounts were generally observed considering raw, steamed and boiled samples, the only notable exception being that of chalinasterol, which was progressively increased when going from raw to steamed and then to boiled broccoli raab samples, as shown also by the stacked column corresponding to boiled broccoli raab in Figure 5. This outcome might be explained with the severe texture modification induced by boiling in the case of broccoli raab, likely enabling the extraction of chalinasterol from portions of vegetal tissue from which the extraction was hindered in the case of the raw product. Notably, boiling is the more common cooking method adopted for the preparation of broccoli raab-based traditional dishes (e.g., *Orecchiette e cime di rapa*) in Southern Italy, where boiled vegetables represent a relevant component of the Mediterranean Diet [87]. If the results obtained for juvenile *Brassica* products are integrated with those obtained from raw or cooked mature kale and broccoli raab, a consistent picture emerges. Data indicate that some juvenile products reach FS concentrations close to those of mature tissues while retaining a more diversified sterol profile. This finding is directly relevant to the working hypothesis of the present study, namely that kale and broccoli raab in early developmental stages can already represent nutritionally meaningful sources of FSs.

In the case of kale, microgreens and microleaves contained sterol amounts comparable to those of cooked mature forms, with the added benefit of a more diversified profile (Figure 3). In broccoli raab, microleaves exhibited the highest total FS concentration among the juvenile products and values only slightly lower than those of cooked mature plants. Therefore, despite their different culinary uses, since they have to be considered ready-to-eat products, juvenile Brassica plants can be considered interesting vegetables also in terms of FS supply. The consumption of 100 g of kale microgreens or broccoli raab microleaves would provide approximately 20–25 mg of total FSs. Not surprisingly, these amounts are lower than total FS amounts typically found in 100 g of vegetal oils, that are usually at least one order of magnitude higher [39]. On the other hand, they are comparable, or even higher than those reported in the literature for several mature fresh vegetables, including cabbage, lettuce, and cauliflower [39]. Considering the advantages associated with the cultivation of juvenile products in terms of sustainability, kale and broccoli raab microgreens and microleaves can thus be regarded as plausible alternatives to the respective adult forms and also to other mature vegetables. Notably, 100 g of kale microgreens or broccoli raab microleaves would be able to provide about 10% of the average daily sterol intake estimated for a non-vegetarian diet [41]. This kind of estimate might also contribute to the development of a Nutritional Quality Score for innovative *Brassicaceae* products, by analogy with recent attempts made to integrate compositional markers into broader quality scores for certain Asteraceae products [88]. On the other hand, if the intake of sterols/stanols that has been reported to be able to significantly reduce cholesterol absorption from the gut (by at least 30%) or plasma LDL cholesterol levels (by at least 8–10%), i.e., 1.6–2 g/day [89] is considered, it is clear that, like most conventional vegetables, non-mature *Brassicaceae* products can represent just one of the components of a diet including vegetables and fruits, to be complemented with richer sources of FSs like vegetal oils.

Apart from these dietary considerations, quantitative data obtained for FSs on the products of interest for the present study support the concept that the developmental stage is an important determinant of sterol accumulation in *Brassica* plant edible tissues and should therefore be considered when assessing the nutritional value of these products.

## 3. Materials and Methods

### 3.1. Chemicals

LC-MS-grade acetonitrile (ACN), water (H_2_O) and formic acid (FA), employed for mobile phase preparation, HPLC-grade chloroform (CHCl_3_) and ethanol (CH_2_CH_3_OH), used as solvents for stock solutions, and HPLC-grade methanol (CH_3_OH), hexane, sodium hydroxide (NaOH), orthophosphoric acid (H_3_PO_4_) (85% *w*/*w*) and sodium chloride (NaCl) used for sterol extraction from *Brassica* vegetables, were purchased from Merck (Milan, Italy). Standards of cholesterol-d_6_ (cholest-5-en-26,26,26,27,27,27-d6-3β-ol), cholesterol (cholest-5-en-3β-ol), campesterol (campest-5-en-3β-ol), β-sitosterol (stigmast-5-en-3β-ol), brassicasterol (ergosta-5,22E-dien-3β-ol), stigmasterol (stigmasta-5,22E-dien-3β-ol), isofucosterol (24Z-ethylidene-cholest-5-en-3β-ol) and Δ^7^-avenasterol (24Z-ethylidene-cholest-7-en-3β-ol) were acquired from Cayman Chemical (Ann Arbor, MI, USA). Standards of lathosterol (cholest-7-en-3β-ol) and chalinasterol (24-methylene-cholest-5-en-3β-ol) were purchased from Merck (Milan, Italy). Specific solutions for the calibration of mass spectrometer under positive or negative polarity conditions were bought from Thermo Scientific (Waltham, MA, USA).

### 3.2. Plant Samples Production

Microgreens, microleaves and baby leaves were cultivated in winter, from 9 January to 9 March 2023, using a hydroponic system in a greenhouse located at the University of Bari Aldo Moro (41°06′ N, 16°52′ E; Southern Italy). Additionally, microgreens and microleaves were cultivated during a spring cycle, from 21 March to 9 May 2023. Notably, winter and spring were selected because they represent the typical cultivation periods for *Brassica* crops under greenhouse conditions in the Mediterranean area. In both cases, the cultivation was conducted in an unheated greenhouse under natural light conditions. Consequently, plants were exposed to the natural seasonal variation in temperature and photoperiod. Temperature trends recorded throughout the experimental period are reported in Supplementary Figure S6. As for the photoperiod, values for the time intervals involved in the two cultivation cycles in 2023 at the location in which the greenhouse was placed were obtained from an online database (www.timeanddate.com) showing an increase from 9 h 24 min (9 January 2023) to 11 h 37 min (9 March 2023) during the winter cycle, and from 12 h 10 min (21 March 2023) to 14 h 16 min (9 May 2023) during the spring one. It is worth noting that light intensity was not continuously monitored during the experiment.

Two different genotypes of *Brassicaceae* were grown: *Brassica rapa* L. subsp. *sylvestris* L. Janch. var. *esculenta* Hort, local variety ‘Cima di rapa novantina’ (broccoli raab); *Brassica oleracea* L. var. *acephala*, local variety ‘Cavolo riccio’ (kale). High-quality seeds, with 95% germination at a constant temperature of 20 °C, were adopted. The experiment was arranged as a split-plot design, where the plant stage (microgreens, microleaves, and baby leaves) was assigned to the main plots and the species (kale and broccoli raab) to the sub-plots. Trays (for microgreens) and pots (for microleaves and baby leaves) for the two species were arranged inside the greenhouse so that they were located in three different sub-sections of the greenhouse. Each sub-section was considered a biological replicate, to account for eventual variabilities related to the different position inside the greenhouse. At the moment of harvesting, plant material was collected from trays or pots located in each sub-section and transferred to the laboratory for sample preparation (see Section 3.3). Specific further details on microgreen, microleaf and baby leaf cultivation are reported in Section S3 of the Supplementary Material.

The same genotypes of kale and broccoli raab were also grown in an open field in Valenzano (41°03′ N 16°53′ E, Southern Italy) from January to March 2023 (winter cycle) to obtain their mature counterparts. In this case, plants were also subjected to cooking by either steaming or boiling. Details on cultivation and cooking procedures are reported in Section S4 of the Supplementary Material.

### 3.3. Sample Preparation

Upon collection at the appropriate growth stages, as described in Section 3.2, all plant material was immediately transferred to the laboratory and frozen at −20 °C. After four days, the material was freeze-dried in a ScanVac CoolSafe 55-9 Pro freeze dryer (LaboGene ApS, Lynge, Denmark) for five days. In particular, a total of 600 microgreens, 10 microleaves and 5 baby leaves, collected for each of the two plant species and related to each of the three greenhouse sub-sections described before, were subjected to lyophilization. In the case of mature vegetables, the material corresponding to three distinct 150 g aliquots of raw kale or broccoli raab, including stems, leaves and inflorescences, was subjected to separate lyophilization. Three aliquots of the same amount were also considered for the two plants subjected to steaming or boiling (see details in Section S4). In all cases, the lyophilized material was manually ground and pooled to obtain one composite sample for each greenhouse sub-section or each 150 g aliquot of raw or cooked mature plant, according to the case, and was then transferred to an individual Falcon tube for storage before extraction and LC-MS analysis. Notably, the lyophilized material associated with a single microgreen, microleaf or baby leaf would have been too limited to enable a reliable extraction and quantification of sterols, thus a pooling of that material, based on each sub-section of the greenhouse from which it came from, was chosen as the best experimental approach. Each Falcon tube containing lyophilized material was stored at −18 °C until FS extraction and analysis were performed, with a single aliquot of material being sampled from each Falcon and subjected to extraction. This last step occurred no later than three days after the transfer of lyophilized material into the Falcon tube, in order to minimize any risk of sterol degradation upon storage.

### 3.4. Sterol Extraction

FSs were extracted from freeze-dried microgreens, microleaves, baby leaves, and mature forms of kale and broccoli raab following the protocol reported by Castellaneta et al. [66], after some modifications. Specifically, the freeze-dried plant samples were further crushed with a mortar, then 50 mg of powder was weighed and 1 mL of CH_3_OH was added; the resulting suspension was sonicated in a water bath for 15 min at 30 °C. To release FSs from sterol SEs and ASGs, basic hydrolysis was performed by adding 2.5 mL of a solution of NaOH in MeOH (1 M) and incubating for 30 min at 60 °C. After cooling to room temperature, the excess base was neutralized by adding 3.5 mL of NaCl in H_2_O (1 M) and 200 μL of aqueous H_3_PO_4_ solution (50% *v*/*v*). The extraction of total FSs, comprising both the ones originally present in the sample and those released through saponification, was then carried out by adding 3.75 mL of hexane and centrifuging the mixture at 5000 rpm for 10 min. After complete phase separation, the supernatant was collected, and the extraction was repeated twice to ensure maximum recovery of FSs. The combined supernatants were then dried under a gentle stream of nitrogen at room temperature. The dry residue was reconstituted in 500 μL of a CH_3_OH/CHCl_3_ solution (2:1 *v*/*v*). Prior to RPLC-APCI(+)-HRMS analysis, the extract was diluted 1:5 (*v*/*v*) with ACN and cholesterol-d_6_ was added as the internal standard at a 10 μg/mL concentration.

### 3.5. Calibration of FS Analytical Response

Stock solutions of each FS standard (1 mg/mL) were prepared in an CH_2_CH_3_OH:CHCl_3_ solution (2:1 *v*/*v*). Calibration was then performed for β-sitosterol, campesterol, brassicasterol, chalinasterol, isofucosterol, Δ^7^-avenasterol, stigmasterol, cholesterol and lathosterol to quantify these sterols in plant samples. Six calibration levels were prepared for each sterol by appropriate dilution in ACN, with values depending on the concentrations expected in real samples: 0.1, 1, 5, 25, 50 and 100 μg/mL for β-sitosterol; 0.1, 1, 5, 15, 25, and 50 μg/mL for campesterol; 0.01, 0.1, 0.5, 1, 5 and 10 μg/mL for brassicasterol, chalinasterol, isofucosterol and Δ^7^-avenasterol; and 0.001, 0.01, 0.05, 0.1, 1, 5 μg/mL for stigmasterol, cholesterol and lathosterol.

### 3.6. RPLC-APCI-HRMS Instrumentation and Operating Conditions

Calibration solutions and plant sample extracts were analyzed using an Ultimate 3000 HPLC system coupled with a Q-Exactive high-resolution quadrupole-Orbitrap mass spectrometer through an APCI interface (Thermo Fisher, West Palm Beach, CA, USA). The RPLC separation of free sterols was achieved using a C18 Ascentis Express HPLC column (150 mm length × 2.1 mm i.d., 2.7 μm particle size) (Supelco, Bellefonte, PA, USA) and the following multistep binary elution gradient, based on H_2_O as phase A and ACN as phase B, both containing 0.1% (*v*/*v*) FA: 0–40 min, linear increase in B from 90% to 100%; 40–50 min, isocratic at 100% B; 50–52 min, linear decrease in B from 100% to 90%; 52−60 min, reconditioning at 90% B. The flow rate was set at 0.250 mL/min and column temperature was kept at 30 °C; 5 μL sample volumes were injected. The parameters of the APCI interface and of the ion optics of the Q-Exactive spectrometer were set as follows: sheath gas flow rate 40 a.u.; auxiliary gas flow rate 10 a.u.; sweep gas flow rate 5 a.u.; discharge current 5 μA; capillary temperature 250 °C; S-lens RF Level 55 a.u.; vaporizer temperature 300 °C. Full MS acquisitions in positive ion mode were performed in a 250–500 *m*/*z* interval at the maximum resolving power of the mass spectrometer (140,000 at *m*/*z* 200); the Automatic Gain Control (AGC) level for the orbital trap filling was set at 1 × 10^6^ and the maximum injection time was 100 ms. HCD-HRMS/MS acquisitions were performed at a 35,000 resolving power in a 50−500 *m*/*z* interval, using a normalized collisional energy (NCE) of 30 units, and selecting a 1.0 *m*/*z* isolation window for precursor ions; the AGC level was set at 2 × 10^5^ and the maximum injection time was 100 ms. The spectrometer was calibrated every two days by infusing, at a 30 μL/min flow rate, calibration solutions for positive or negative polarity acquisitions, thus obtaining a mass accuracy always better than 5 ppm.

## Figures and Tables

**Table 1:** Concentrations of free sterols (mg/100 g fresh weight, FW) obtained by RPLC-APCI-HRMS analysis of extracts of microgreens, microleaves, baby leaves and mature plants of kale and broccoli raab. Data represent 95% confidence intervals for mean concentrations (*n* = 3). Superscript letters represent the outcome of one-way ANOVA with Tukey’s HSD post hoc test (*p* = 0.05), performed for each sterol across the different products, i.e., for the comparison between data reported in each row.

**Compound**	**Kale**			
	**Micro-** **Greens**	**Micro-** **Leaves**	**Baby** **Leaves**	**Mature** **Plants**
β-sitosterol	18 ± 2 ^ab^	18 ± 2 ^ab^	13 ± 2 ^b^	23 ± 6 ^a^
Campesterol	3.4 ± 0.3 ^a^	2.6 ± 0.3 ^b^	1.7 ± 0.3 ^c^	2.6 ± 0.5 ^b^
Isofucosterol	0.51 ± 0.15 ^a^	0.33 ± 0.05 ^b^	0.30 ± 0.05 ^b^	0.16 ± 0.05 ^c^
Brassicasterol	0.42 ± 0.18	<LOD	<LOD	<LOD
Cholesterol	0.27 ± 0.04 ^a^	0.044 ± 0.016 ^b^	0.052 ± 0.011 ^b^	<LOD
Chalinasterol	0.20 ± 0.05 ^a^	0.122 ± 0.007 ^b^	0.11 ± 0.03 ^b^	<LOD
24-epibrassicasterol	0.19 ± 0.11	<LOD	<LOD	<LOD
Δ^7^-avenasterol	0.14 ± 0.03 ^a^	0.11 ± 0.03 ^a^	0.13 ± 0.03 ^a^	0.15 ± 0.05 ^a^
**Compound**	**Broccoli Raab**			
	**Micro-** **Greens**	**Micro** **Leaves**	**Baby** **Leaves**	**Mature** **Plants**
β-sitosterol	10.3 ± 1.1 ^b^	18 ± 4 ^ab^	13 ± 5 ^ab^	21 ± 9 ^a^
Campesterol	1.61 ± 0.16 ^a^	2.0 ± 0.2 ^a^	1.7 ± 0.5 ^a^	2.1 ± 0.6 ^a^
Isofucosterol	0.42 ± 0.05 ^b^	0.8 ± 0.2 ^a^	0.51 ± 0.18 ^b^	0.4 ± 0.2 ^b^
Brassicasterol	0.36 ± 0.07	<LOD	<LOD	<LOD
Cholesterol	0.21 ± 0.02 ^b^	0.30 ± 0.09 ^b^	0.21 ± 0.09 ^b^	0.6 ± 0.3 ^a^
Chalinasterol	0.115 ± 0.016 ^b^	0.23 ± 0.06 ^a^	0.23 ± 0.07 ^a^	0.17 ± 0.07 ^ab^
24-epibrassicasterol	0.09 ± 0.02	<LOD	<LOD	<LOD
Δ^7^-avenasterol	0.062 ± 0.009 ^a^	0.10 ± 0.03 ^a^	0.11 ± 0.04 ^a^	0.12 ± 0.08 ^a^

## Data Availability

The raw data supporting the conclusions of this article will be made available by the authors on request.

## Supplementary Materials

The following supporting information can be downloaded at https://www.mdpi.com/article/10.3390/molecules31142553/s1, Section S1: Inference of structural information on minor free sterols detected in the extracts of kale and broccoli raab products; Section S2: Evaluation of analytical response variabilities related to instrumental fluctuations or to sampling of freeze-dried vegetal samples combined with extraction yield reproducibility; Section S3: Details on the cultivation of kale and broccoli raab microgreens, microleaves and baby leaves; Section S4: Details on the cultivation and cooking of mature forms of kale and broccoli raab; Table S1: Summary of data concerning the external calibration of standard sterols: Concentration range, calibration line equation and coefficient of determination (R2). The LOD and LOQ values (μg/mL) were determined as 3 and 10 times the ratio of the calibration intercept standard deviation to the slope, respectively; Table S2: Percent relative standard deviation (RSD) values related to the sampling-extraction variability and the analytical response variability evaluated for standard sterols and for 24-epibrassicasterol in kale microgreens. Sampling-extraction variability was estimated from the extraction and LC-MS analysis of five separate aliquots sampled from the same batch of lyophilized material (corresponding to a specific sub-section of the greenhouse in which kale microgreens were cultivated) processed on the same day. The analytical response variability was assessed by repeating the LC-MS analysis of a single extract three times on alternate days within a week, with the extract being stored at −18 °C between the analyses; Table S3: Free sterol contents (mg/100 g FW) of mature kale and broccoli raab after steaming or boiling in water. Values represent 95% confidence intervals centred on means (*n* = 3); Figure S1: APCI(+)-HCD-HRMS/MS spectra acquired for the [M + H − H_2_O]^+^ ions of sterols whose peaks were detected at specific retention times in the EIC traces referred to exact *m*/*z* values 381.3516 (plots (A–C)) and 395.3672 (plots (D,E)) upon RPLC-APCI-HRMS analysis of a kale microgreens extract (see Figure 2). Peak signals of product ions sharing the same number of carbon atoms but differing in hydrogen atom count were grouped into clusters, each identified by a capital letter. The experimental *m*/*z* value of the most intense signal within each cluster is reported; Figure S2: Proposed fragmentation mechanism for the generation of a product ion with exact *m*/*z* 83.0855, consistent with one of the peak signals detected in the APCI(+)-HCD-HRMS/MS spectrum of the sterol eluting at 27.2 min in the EIC chromatogram obtained for *m*/*z* 381.3516 upon RPLC-APCI(+)-HRMS analysis of a kale microgreen extract (Figure 2C). The sterol was tentatively identified as 24-methylcholesta-5,23E-dien-3β-ol, based on its MS/MS profile (see main text for details). Exact monoisotopic *m*/*z* ratios, rounded off to the fourth decimal place, are reported; Figure S3: Proposed fragmentation mechanism for the generation of a product ion with exact *m*/*z* 255.2107, consistent with one of the peak signals detected in the APCI(+)-HCD-HRMS/MS spectrum of the sterol eluting at 30.2 min in the EIC chromatogram obtained for *m*/*z* 395.3672 upon RPLC-APCI(+)-HRMS analysis of a kale microgreen extract (Figure 2D). The sterol was tentatively identified as Δ^5,24^-stigmastadienol, based on its MS/MS spectrum. Exact monoisotopic *m*/*z* ratios, rounded off to the fourth decimal place, are reported; Figure S4: Stacked-bar graph reporting the average cumulative percentual recovery (*n* = 3) of main sterols detected in kale microgreens evaluated after each of three successive extraction steps; Figure S5: Column graphs showing average free sterol concentrations (mg/100 g FW) found in microgreens and microleaves of kale and broccoli raab grown in winter (blue bars) and spring (green bars). Error bars represent 95% confidence intervals (*n* = 3 for each product type). Asterisks indicate statistically significant differences between the two seasons (*p* < 0.05); Figure S6: Average, minimum and maximum temperatures recorded inside the greenhouse during the two growing cycles (winter: 9 January–9 March 2023; spring: 21 March–9 May 2023) adopted for the cultivation of microgreens and microleaves of kale and broccoli raab.

## References

[1] Di Gioia F., Renna M., Santamaria P. (2017). Sprouts, Microgreens and “Baby Leaf” Vegetables. Minimally Processed Refrigerated Fruits and Vegetables.

[2] Renna M., Paradiso V.M. (2020). Ongoing Research on Microgreens: Nutritional Properties, Shelf-Life, Sustainable Production, Innovative Growing and Processing Approaches. Foods.

[3] Ebert A.W. (2022). Sprouts and Microgreens—Novel Food Sources for Healthy Diets. Plants.

[4] Sharma S., Shree B., Sharma D., Kumar S., Kumar V., Sharma R., Saini R. (2022). Vegetable Microgreens: The Gleam of next Generation Super Foods, Their Genetic Enhancement, Health Benefits and Processing Approaches. Food Res. Int..

[5] Partap M., Sharma D., Hn D., Thakur M., Verma V., Ujala, Bhargava B. (2023). Microgreen: A Tiny Plant with Superfood Potential. J. Funct. Foods.

[6] Choe U., Yu L.L., Wang T.T.Y. (2018). The Science behind Microgreens as an Exciting New Food for the 21st Century. J. Agric. Food Chem..

[7] Teng J., Liao P., Wang M. (2021). The Role of Emerging Micro-Scale Vegetables in Human Diet and Health Benefits—An Updated Review Based on Microgreens. Food Funct..

[8] Treadwell D.D., Hochmuth R., Landrum L., Laughlin W. (2020). Microgreens: A New Specialty Crop: HS1164, Rev. 9/2020.

[9] Xiao Z., Lester G.E., Luo Y., Wang Q. (2012). Assessment of Vitamin and Carotenoid Concentrations of Emerging Food Products: Edible Microgreens. J. Agric. Food Chem..

[10] Aires A. (2018). Hydroponic Production Systems: Impact on Nutritional Status and Bioactive Compounds of Fresh Vegetables.

[11] Sambo P., Nicoletto C., Giro A., Pii Y., Valentinuzzi F., Mimmo T., Lugli P., Orzes G., Mazzetto F., Astolfi S. (2019). Hydroponic Solutions for Soilless Production Systems: Issues and Opportunities in a Smart Agriculture Perspective. Front. Plant Sci..

[12] Lenzi A., Orlandini A., Bulgari R., Ferrante A., Bruschi P. (2019). Antioxidant and Mineral Composition of Three Wild Leafy Species: A Comparison between Microgreens and Baby Greens. Foods.

[13] Kyriacou M.C., Rouphael Y., Di Gioia F., Kyratzis A., Serio F., Renna M., De Pascale S., Santamaria P. (2016). Micro-Scale Vegetable Production and the Rise of Microgreens. Trends Food Sci. Technol..

[14] Renna M., Castellino M., Leoni B., Paradiso V.M., Santamaria P. (2018). Microgreens Production with Low Potassium Content for Patients with Impaired Kidney Function. Nutrients.

[15] Di Gioia F., Petropoulos S.A., Ozores-Hampton M., Morgan K., Rosskopf E.N. (2019). Zinc and Iron Agronomic Biofortification of Brassicaceae Microgreens. Agronomy.

[16] Pannico A., El-Nakhel C., Graziani G., Kyriacou M.C., Giordano M., Soteriou G.A., Zarrelli A., Ritieni A., De Pascale S., Rouphael Y. (2020). Selenium Biofortification Impacts the Nutritive Value, Polyphenolic Content, and Bioactive Constitution of Variable Microgreens Genotypes. Antioxidants.

[17] Puccinelli M., Pezzarossa B., Pintimalli L., Malorgio F. (2021). Selenium Biofortification of Three Wild Species, *Rumex acetosa* L., *Plantago coronopus* L., and *Portulaca oleracea* L., Grown as Microgreens. Agronomy.

[18] Zhang X., Bian Z., Yuan X., Chen X., Lu C. (2020). A Review on the Effects of Light-Emitting Diode (LED) Light on the Nutrients of Sprouts and Microgreens. Trends Food Sci. Technol..

[19] Alrifai O., Hao X., Marcone M.F., Tsao R. (2019). Current Review of the Modulatory Effects of LED Lights on Photosynthesis of Secondary Metabolites and Future Perspectives of Microgreen Vegetables. J. Agric. Food Chem..

[20] Samuoliene G., Brazaityte A., Sirtautas R., Sakalauskiene S., Jankauskiene J., Duchovskis P., Novičkovas A. (2012). The Impact of Supplementary Short-Term Red LED Lighting on the Antioxidant Properties of Microgreens. Acta Hortic..

[21] Samuoliene G., Brazaityte A., Jankauskiene J., Viršile A., Sirtautas R., Novičkovas A., Sakalauskiene S., Sakalauskaite J., Duchovskis P. (2013). LED Irradiance Level Affects Growth and Nutritional Quality of Brassica Microgreens. Cent. Eur. J. Biol..

[22] Brazaitytė A., Miliauskienė J., Vaštakaitė-Kairienė V., Sutulienė R., Laužikė K., Duchovskis P., Małek S. (2021). Effect of Different Ratios of Blue and Red Led Light on Brassicaceae Microgreens under a Controlled Environment. Plants.

[23] Demir K., Sarıkamış G., Çakırer Seyrek G. (2023). Effect of LED Lights on the Growth, Nutritional Quality and Glucosinolate Content of Broccoli, Cabbage and Radish Microgreens. Food Chem..

[24] Samuolienė G., Viršilė A., Brazaitytė A., Jankauskienė J., Sakalauskienė S., Vaštakaitė V., Novičkovas A., Viškelienė A., Sasnauskas A., Duchovskis P. (2017). Blue Light Dosage Affects Carotenoids and Tocopherols in Microgreens. Food Chem..

[25] Martínez-Ispizua E., Calatayud Á., Marsal J.I., Cannata C., Basile F., Abdelkhalik A., Soler S., Valcárcel J.V., Martínez-Cuenca M.R. (2022). The Nutritional Quality Potential of Microgreens, Baby Leaves, and Adult Lettuce: An Underexploited Nutraceutical Source. Foods.

[26] Martínez-Sánchez A., Gil-Izquierdo A., Gil M.I., Ferreres F. (2008). A Comparative Study of Flavonoid Compounds, Vitamin C, and Antioxidant Properties of Baby Leaf *Brassicaceae* Species. J. Agric. Food Chem..

[27] Waterland N.L., Moon Y., Tou J.C., Kim M.J., Pena-Yewtukhiw E.M., Park S. (2017). Mineral Content Differs among Microgreen, Baby Leaf, and Adult Stages in Three Cultivars of Kale. HortScience.

[28] Aires A., Carvalho R., Rosa E.A.S., Saavedra M.J. (2013). Phytochemical Characterization and Antioxidant Properties of Baby-Leaf Watercress Produced under Organic Production System. CyTA—J. Food.

[29] Saini R.K., Ko E.Y., Keum Y.S. (2017). Minimally Processed Ready-to-Eat Baby-Leaf Vegetables: Production, Processing, Storage, Microbial Safety, and Nutritional Potential. Food Rev. Int..

[30] Ramirez D., Abellán-Victorio A., Beretta V., Camargo A., Moreno D.A. (2020). Functional Ingredients from *Brassicaceae* Species: Overview and Perspectives. Int. J. Mol. Sci..

[31] Le T.N., Chiu C.-H., Hsieh P.-C. (2020). Bioactive Compounds and Bioactivities of *Brassica oleracea* L. Var. Italica Sprouts and Microgreens: An Updated Overview from a Nutraceutical Perspective. Plants.

[32] Vučetić A., Šovljanski O., Pezo L., Gligorijević N., Kostić S., Vulić J., Čanadanović-Brunet J. (2025). A Comprehensive Antioxidant and Nutritional Profiling of Brassicaceae Microgreens. Antioxidants.

[33] Bafumo R.F., Alloggia F.P., Ramirez D.A., Maza M.A., Camargo A.B., Ramirez D.A., Maza M.A., Fontana A., Moreno D.A., Camargo A.B. (2024). Optimal Brassicaceae Family Microgreens from a Phytochemical and Sensory Perspective. Food Res. Int..

[34] Xiao Z., Rausch S.R., Luo Y., Sun J., Yu L., Wang Q., Chen P., Yu L., Stommel J.R. (2019). Microgreens of Brassicaceae: Genetic Diversity of Phytochemical Concentrations and Antioxidant Capacity. LWT—Food Sci. Technol..

[35] Liu Z., Shi J., Wan J., Pham Q., Zhang Z., Sun J., Yu L., Luo Y., Wang T.T.Y., Chen P. (2022). Profiling of Polyphenols and Glucosinolates in Kale and Broccoli Microgreens Grown under Chamber and Windowsill Conditions by Ultrahigh-Performance Liquid Chromatography High-Resolution Mass Spectrometry. ACS Food Sci. Technol..

[36] Sun J., Xiao Z., Lin L.Z., Lester G.E., Wang Q., Harnly J.M., Chen P. (2013). Profiling Polyphenols in Five Brassica Species Microgreens by UHPLC-PDA-ESI/HRMSn. J. Agric. Food Chem..

[37] Castellaneta A., Losito I., Cisternino G., Leoni B., Santamaria P., Calvano C.D., Bianco G., Cataldi T.R.I. (2022). All Ion Fragmentation Analysis Enhances the Untargeted Profiling of Glucosinolates in Brassica Microgreens by Liquid Chromatography and High-Resolution Mass Spectrometry. J. Am. Soc. Mass Spectrom..

[38] Di Bella M.C., Toscano S., Arena D., Moreno D.A., Romano D., Branca F. (2021). Effects of Growing Cycle and Genotype on the Morphometric Properties and Glucosinolates Amount and Profile of Sprouts, Microgreens and Baby Leaves of Broccoli (*Brassica oleracea* L. var. *italica* Plenck) and Kale (*B. oleracea* L. var. *acephala* DC.). Agronomy.

[39] Poudel P., Petropoulos S.A., Di Gioia F., Carocho M., Heleno S.A., Barros L. (2023). Plant Tocopherols and Phytosterols and Their Bioactive Properties. Natural Secondary Metabolites.

[40] De Jong A., Plat J., Mensink R.P. (2003). Metabolic Effects of Plant Sterols and Stanols (Review). J. Nutr. Biochem..

[41] Piironen V., Lindsay D.G., Miettinen T.A., Toivo J., Lampi A.M. (2000). Plant Sterols: Biosynthesis, Biological Function and Their Importance to Human Nutrition. J. Sci. Food Agric..

[42] Mongrand S., Stanislas T., Bayer E.M.F., Lherminier J., Simon-Plas F. (2010). Membrane Rafts in Plant Cells. Trends Plant Sci..

[43] Valitova J.N., Sulkarnayeva A.G., Minibayeva F.V. (2016). Plant Sterols: Diversity, Biosynthesis, and Physiological Functions. Biochemistry.

[44] Moreau R.A., Nyström L., Whitaker B.D., Winkler-Moser J.K., Baer D.J., Gebauer S.K., Hicks K.B. (2018). Phytosterols and Their Derivatives: Structural Diversity, Distribution, Metabolism, Analysis, and Health-Promoting Uses. Prog. Lipid Res..

[45] Simon-Plas F., Perraki A., Bayer E., Gerbeau-Pissot P., Mongrand S. (2011). An Update on Plant Membrane Rafts. Curr. Opin. Plant Biol..

[46] Du Y., Fu X., Chu Y., Wu P., Liu Y., Ma L., Tian H., Zhu B. (2022). Biosynthesis and the Roles of Plant Sterols in Development and Stress Responses. Int. J. Mol. Sci..

[47] Schaller H. (2003). The Role of Sterols in Plant Growth and Development. Prog. Lipid Res..

[48] Rogowska A., Szakiel A. (2020). The Role of Sterols in Plant Response to Abiotic Stress. Phytochem. Rev..

[49] Ferrer A., Altabella T., Arró M., Boronat A. (2017). Emerging Roles for Conjugated Sterols in Plants. Prog. Lipid Res..

[50] Moreau R.A., Whitaker B.D., Hicks K.B. (2002). Phytosterols, Phytostanols, and Their Conjugates in Foods: Structural Diversity, Quantitative Analysis, and Health-Promoting Uses. Prog. Lipid Res..

[51] Münger L.H., Jutzi S., Lampi A.M., Nyström L. (2015). Comparison of Enzymatic Hydrolysis and Acid Hydrolysis of Sterol Glycosides from Foods Rich in Δ^7^-Sterols. Lipids.

[52] Münger L.H., Nyström L. (2014). Enzymatic Hydrolysis of Steryl Glycosides for Their Analysis in Foods. Food Chem..

[53] Evtyugin D.D., Evtuguin D.V., Casal S., Domingues M.R. (2023). Advances and Challenges in Plant Sterol Research: Fundamentals, Analysis, Applications and Production. Molecules.

[54] Hartmann M.A., Benveniste P. (1987). Plant Membrane Sterols: Isolation, Identification, and Biosynthesis. Methods Enzymol..

[55] Moss G.P. (1989). Nomenclature of Steroids (Recommendations 1989). Pure Appl. Chem..

[56] Schlag S., Vetter W. (2024). Quantitative Data of up to Thirty Sterols in Vegetable Oils and Fats. Eur. Food Res. Technol..

[57] Nes W.D. (2011). Biosynthesis of Cholesterol and Other Sterols. Chem. Rev..

[58] Gajewski M., Przybył J.L., Szymczak P. (2007). Broccoli as the Source of Phytosterols in Nutrition. Bull. Univ. Agric. Sci. Vet. Med. Cluj-Napoca.

[59] Gajewski M., PrzybyŁ J.L., Kosakowska O., Szymczak P. (2009). Some Factors Influencing Free Sterols Content in Broccoli (*Brassica oleracea* L. var. *Botrytis italica* Plenck.). J. Food Biochem..

[60] Baek S.A., Jung Y.H., Lim S.H., Park S.U., Kim J.K. (2016). Metabolic Profiling in Chinese Cabbage (*Brassica rapa* L. subsp. *pekinensis*) Cultivars Reveals That Glucosinolate Content Is Correlated with Carotenoid Content. J. Agric. Food Chem..

[61] Han J.H., Yang Y.X., Feng M.Y. (2008). Contents of Phytosterols in Vegetables and Fruits Commonly Consumed in China. Biomed. Environ. Sci..

[62] Kaloustian J., Alhanout K., Amiot-Carlin M.J., Lairon D., Portugal H., Nicolay A. (2008). Effect of Water Cooking on Free Phytosterol Levels in Beans and Vegetables. Food Chem..

[63] Lee K.B., Kim Y.J., Kim H.J., Choi J., Kim J.K. (2018). Phytochemical Profiles of Brassicaceae Vegetables and Their Multivariate Characterization Using Chemometrics. Appl. Biol. Chem..

[64] Piironen V., Toivo J., Puupponen-Pimiä R., Lampi A.M. (2003). Plant Sterols in Vegetables, Fruits and Berries. J. Sci. Food Agric..

[65] Normen L., Johnsson M., Andersson H., van Gameren Y., Dutta P. (1999). Plant Sterols in Vegetables and Fruits Commonly Consumed in Sweden. Eur. J. Nutr..

[66] Castellaneta A., Losito I., Leoni B., Renna M., Mininni C., Santamaria P., Calvano C.D., Cataldi T.R.I., Liebisch G., Matysik S. (2023). A Targeted GC-MS/MS Approach for the Determination of Eight Sterols in Microgreen and Mature Plant Material. J. Steroid Biochem. Mol. Biol..

[67] Cinquepalmi V., Losito I., Castellaneta A., Calvano C.D., Cataldi T.R.I. (2025). APCI-Multistage Mass Spectrometry Following Liquid Chromatography for Selected 4-Desmethyl-Sterols and Their Deuterium-Labelled Analogues Unveils Characteristic Fragmentation Routes for Cholesterol and Phytosterols Identification. Rapid Commun. Mass. Spectrom..

[68] Cinquepalmi V., Losito I., Castellaneta A., Calvano C.D., Cataldi T.R.I. (2025). Diagnostic Fragmentations of Animal and Fungal Sterols/Stanols Obtained by APCI–Tandem Mass Spectrometry: A Route Towards Unknown Free Sterol Identification. Metabolites.

[69] Sonawane P.D., Pollier J., Panda S., Szymanski J., Massalha H., Yona M., Unger T., Malitsky S., Arendt P., Pauwels L. (2016). Plant Cholesterol Biosynthetic Pathway Overlaps with Phytosterol Metabolism. Nat. Plants.

[70] Münger L.H., Boulos S., Nyström L. (2018). UPLC-MS/MS Based Identification of Dietary Steryl Glucosides by Investigation of Corresponding Free Sterols. Front. Chem..

[71] Kikuchi T., Kadota S., Shima T., Ikekawa N., Fujimoto Y. (1984). Effective Separation of Sterol C-24 Epimers by Reversed-Phase High Performance Liquid Chromatography. Chem. Pharm. Bull..

[72] Ikekawa N., Fujimoto Y., Kadota S., Kikuchi T. (1989). Effective Separation of Sterol C-24 Epimers. J. Chromatogr. A.

[73] Chitwood D.J., Patterson G.W. (1991). Separation of Epimeric Pairs of C-24 Alkylsterols by Reversed-Phase High Performance Liquid Chromatography of the Free Sterols at Subambient Temperature. J. Liq. Chromatogr..

[74] Akihisa T., Ghosh P., Thakur S., Rosentein F.U., Matsumoto T. (1986). Sterol Compositions of Seeds and Mature Plants of Family Cucurbitaceae. J. Am. Oil Chem. Soc..

[75] Akihisa T., Thakur S., Rosenstein F.U., Matsumoto T. (1986). Sterols of Cucurbitaceae: The Configurations at C-24 of 24-Alkyl-Δ^5^-, Δ^7^- and Δ^8^-Sterols. Lipids.

[76] Garg V.K., Nes W.R. (1986). Occurrence of Δ^5^-Sterols in Plants Producing Predominantly Δ^7^-Sterols: Studies on the Sterol Compositions of Six Cucurbitaceae Seeds. Phytochemistry.

[77] Bolker H.I. (1967). Crinosterol: A Unique Sterol from a Comatulid Crinoid. Nature.

[78] Griffiths K.M., Bacic A., Howlett B.J. (2003). Sterol Composition of Mycelia of the Plant Pathogenic Ascomycete Leptosphaeria Maculans. Phytochemistry.

[79] Aoyama Y., Yoshida Y., Sato R. (1984). Yeast Cytochrome P-450 Catalyzing Lanosterol 14α-Demethylation. II. Lanosterol Metabolism by Purified P-450(14DM) and by Intact Microsomes. J. Biol. Chem..

[80] Misso N.L.A., Goad L.J. (1984). Investigations on the Δ^23^-, Δ^24(28)^- and Δ^25^-Sterols of Zea Mays. Phytochemistry.

[81] Kim S.K., Akihisa T., Tamura T., Matsumoto T., Yokota T., Takahashi N. (1988). 24-Methylene-25-Methylcholesterol in *Phaseolus vulgaris* Seed: Structural Relation to Brassinosteroids. Phytochemistry.

[82] Shepherd T., Dobson G., Verrall S.R., Conner S., Griffiths D.W., McNicol J.W., Davies H.V., Stewart D. (2007). Potato Metabolomics by GC-MS: What Are the Limiting Factors?. Metabolomics.

[83] Kruve A., Rebane R., Kipper K., Oldekop M.L., Evard H., Herodes K., Ravio P., Leito I. (2015). Tutorial Review on Validation of Liquid Chromatography-Mass Spectrometry Methods: Part I. Anal. Chim. Acta.

[84] Lu B., Ren Y., Zhang Y., Gong J. (2008). Effects of genetic variability, parts and seasons on the sterol content and composition in bamboo shoots. Food Chem..

[85] Roche J., Alignan M., Bouniols A., Cerny M., Mouloungui Z., Vear F., Merah O. (2010). Sterol content in sunflower seeds (*Helianthus annuus* L.) as affected by genotypes and environmental conditions. Food Chem..

[86] Morikawa T., Mizutani M., Aoki N., Watanabe B., Saga H., Saito S., Oikawa A., Suzuki H., Sakurai N., Shibata D. (2006). Cytochrome P450 *CYP710A* Encodes the Sterol C-22 Desaturase in *Arabidopsis* and Tomato. Plant Cell.

[87] Renna M., Rinaldi V.A., Gonnella M. (2015). The Mediterranean Diet between traditional foods and human health: The culinary example of Puglia (Southern Italy). Int. J. Gastron. Food Sci..

[88] Renna M., Somma A., Leoni B., Castellaneta A., Cinquepalmi V., Losito I., Cataldi T.R.I., Santamaria P. (2025). Globe artichoke’s offshoots: From by-product to new horticultural product. Acta Hortic..

[89] Marangoni F., Poli A. (2010). Phytosterols and cardiovascular health. Pharmacol. Res..

