# Supplementary material for: Free Sterol Content of Brassica Microgreens, Microleaves and Baby Leaves: A Quantitative Study by RPLC-APCI-HRMS
Source: Molecules. 2026 Jul 22;31(14):2553. doi: 10.3390/molecules31142553 (PMC13414607; doi:10.3390/molecules31142553)
Supplement: Supplementary file 1 [file molecules-31-02553-s001.zip › molecules-4407754-supplementary.pdf]

Supplementary Materials

Free Sterol Content of *Brassica* Microgreens, Microleaves and Baby Leaves: A Quantitative Study by RPLC-APCI-HRMS

Valeria Cinquepalmi [1], Ilario Losito [1,2,*], Andrea Castellaneta [1], Beniamino Leoni [3], Massimiliano Renna [3], Onofrio Davide Palmitessa [3], Pietro Santamaria [2,3], Cosima Damiana Calvano [1,2] and Tommaso R. I. Cataldi [1,2]

[1] Dipartimento di Chimica, Università degli Studi di Bari Aldo Moro, Via Orabona 4, 70126 Bari, Italy
[2] Centro Interdipartimentale SMART, Università degli Studi di Bari Aldo Moro, Via Orabona 4, 70126 Bari, Italy
[3] Dipartimento di Scienze del Suolo, della Pianta e degli Alimenti, Università degli Studi di Bari Aldo Moro, Via G. Amendola 165/a, 70126 Bari, Italy
* Correspondence: ilario.losito@uniba.it; Tel.: +39-080-5442506

Sections

Section S1. Inference of structural information on minor free sterols detected in the extracts of kale and broccoli raab products

Structural information on minor free sterols detected in kale and broccoli raab products using RPLC-APCI(+)-HRMS (see Figure 2 in the manuscript) was inferred considering HCD-MS/MS spectra averaged under the respective chromatographic peaks, which are reported in Figure S1 in these Supplementary Materials (*vide infra*). By analogy with our previous studies (Refs. 67 and 68 in the manuscript), signals of ions sharing the same number of carbon atoms and differing only in hydrogen content were grouped within a single cluster, designated by a capital letter.

The species corresponding to an exact *m/z* 381.3516 and eluting after 24.9 min exhibited a 0.8 min shift towards lower retention times compared to chalinasterol. By analogy with the chromatographic behaviour of sterol pairs lathosterol/cholesterol and Δ^7-avenasterol/isofucosterol, this feature suggested that it might correspond to the Δ^7 analogue of chalinasterol. This hypothesis was supported by the corresponding HCD-MS/MS spectrum (Figure S1A). Indeed, the high intensity observed in the respective clusters for product ions detected at *m/z* 69.0708 (cluster B), 149.1323 (cluster H), 161.1321 (cluster I) and 297.2568 (cluster S) was consistent with the typical fragmentation pattern of Δ^7-sterols (Refs. 67,68,70). Moreover, the intensity profiles of clusters J-R and T-U, which are diagnostic for the position of the C=C double bond on the side chain (Refs 67,68), matched those found in the HCD-MS/MS spectrum of chalinasterol (Ref. 68), thereby confirming its location between C24 and C24'. Notably, a Δ^7 analogue of chalinasterol, known as episterol (24-methylene-cholest-7-en-3β-ol), emerged from the search in the LipidMaps database.

The HCD-MS/MS spectrum obtained for the sterol generating an *m/z* 381.3516 ion in the APCI source and eluting after 27.2 min (Figure 2C) is shown in Figure S1B. Considering the effect of the side chain C=C bond on the retention on the C18 stationary phase, evidenced in our previous studies on sterols, the elution of this sterol between chalinasterol and brassicasterol, which include a C=C

bond between C24 and C24' and between C22 and C23, respectively, suggested the presence of a C=C bond between C23 and C24. The structure of 24-methylcholesta-5,23E-dien-3β-ol, reported in the LipidMaps database, is consistent with this hypothesis. The relevant intensity of product ions at *m/z* 147.1166 and 161.1321 in the respective clusters (H and I) of the HCD-MS/MS spectrum (Figure S1B) supported the presence of a C=C bond between C5 and C6 on ring B (Refs. 67 and 68). Additionally, the slight predominance of the *m/z* 83.0861 ion over the one at *m/z* 81.0705 in cluster C was consistent with the occurrence of a fragmentation pathway ultimately leading to an allylic carbocation (exact *m/z* 83.0855) and favoured by the presence of a C=C bond between C23 and C24, as depicted in Figure S2.

The last chromatographic peak observed in the EIC trace for *m/z* 381.3516 and not identifiable through comparison with standards was eluted after 30.9 min, *i.e.*, close to that of brassicasterol (Figure 2C). This behaviour suggested a strong structural similarity with the latter at the level of the side chain; thus, the sterol was tentatively identified as the α epimer of brassicasterol, which is listed in the LipidMaps database as campesta-5,22E-dien-3β-ol, also known as 24-epibrassicasterol or crinosterol. The hypothesis was confirmed by the remarkable similarity observed between the HCD-MS/MS spectrum of the unknown sterol (Figure S1C) and that obtained for brassicasterol (see Figure 4 in Ref. 67). Notably, nearly identical HCD-MS/MS spectra were also observed for the epimeric stanols cholestanol and coprostanol during our recent study on animal/fungal sterols (Ref. 68). Furthermore, previous studies indicated that α epimers of sterols are eluted before the corresponding β epimers on a C18 stationary phase (see Refs. 71–73).

Turning to the EIC chromatogram obtained for *m/z* 395.3672 (Figure 2D), two peaks not corresponding to any of the available sterol standards were considered for a tentative identification. The peak eluting at 29.2 min exhibited the HCD-MS/MS spectrum shown in Figure S1D. Diagnostic ions recently identified for 4,4-dimethyl-Δ^8-sterols (Ref. 68) considering lanosterol as a model, such as those prevailing in clusters H, I, K, M and N, at *m/z* 149.1323, 163.1475, 191.1797, 217.1954 and 231.2110, respectively, were clearly detected. In contrast to lanosterol, no peak signal at *m/z* 339.3052

(*i.e.*, in cluster V), which would correspond to the carbocation of a Δ^8 steroidal backbone methylated twice on C4 and once on C14, was observed for the unknown sterol. Regarding the side chain of the unknown sterol, product ions prevailing in clusters J-S indicated the presence of a C=C bond between C24 and C25, based on recently described fragmentation trends (Ref. 68). By combining the information retrieved from the HCD-MS/MS spectrum with a search on the LipidMaps database, based on the *m/z* value found for its $[M + H - H_2O]^+$ ion, the sterol eluting at 29.2 min was tentatively identified as 14-demethyl-lanosterol, also known as 4,4-dimethylzymosterol or 4,4-dimethyl-5α-cholesta-8,24-dien-3β-ol. This assignment was also supported chromatographically: comparison with retention times previously observed under identical conditions for zymosterol and lanosterol (Ref. 68) indicated that 14-demethyl-lanosterol is eluted between these sterols. This finding reflects the retention order Δ^8-sterol < 4,4-dimethyl-Δ^8-sterol < 4,4,14-trimethyl-Δ^8-sterol, which is consistent with the increased hydrophobicity resulting from additional methylations on the steroidal backbone.

The final unidentified peak in the EIC chromatogram for *m/z* 395.3672 was eluted at 30.2 min (Figure 2D). The corresponding HCD-MS/MS spectrum (Figure S1E) exhibited prevailing product ions in clusters H and I at *m/z* 147.1166 and 161.1321, respectively, a feature previously recognized for Δ^5-sterols (Refs 67-70). A key for identifying the side chain was provided by the prevailing ion in cluster T, at *m/z* 313.2880. This feature was not consistent with the side chain structure of stigmasterol and isofucosterol (Ref. 67), including an ethyl moiety linked to C24 and a C=C bond (in the former case located within that moiety), although the *m/z* value of the precursor ion and the detection of prevailing product ions at *m/z* 325.2890 and 339.3046 in clusters U and V, respectively, were consistent with them. $\Delta^{5,24}$-stigmastadienol, which includes an ethyl moiety at C24 and a C=C bond between C24 and C25, was therefore considered as a possible assignment for the unknown sterol. This hypothesis was confirmed by the clear predominance of the *m/z* 255.2107 ion in cluster P (Figure S1E), a feature differing from those previously found for other sterols in this cluster (Refs. 67,68). As illustrated in Figure S3, the concurrent presence of an ethyl moiety at C24 and a C=C bond between C24 and C25

can facilitate a specific fragmentation pathway leading to a product ion with an exact *m/z* 255.2107, involving a 1,5 H-transfer from C16 to C23.

Section S2. Evaluation of analytical response variabilities related to instrumental fluctuations or to sampling of freeze-dried vegetal samples combined with extraction yield reproducibility.

In order to evaluate the analytical response variability related to instrumental fluctuations, a single extract obtained from an aliquot of freeze-dried kale microgreens was analysed by RPLC-APCI-HRMS on alternate days over a five-day period, with storage at –18 °C between analyses. The evaluation focused on sterols whose concentrations were higher than LOD values, thus excluding sterols having low concentrations, that were detected only by considering chromatographic and MS/MS data (excepting 24-epibrassicasterol) and lathosterol and stigmasterol, that could not be quantified in kale microgreens due to low concentration. As shown in the third column of Table S2 in these Supplementary Materials (*vide infra*), the relative standard deviations (RSDs) of normalized EIC peak areas obtained for selected sterols were excellent (< 6%), confirming the efficacy of response normalization based on the isotopically labelled internal standard and the stability of sterols in extracts over the 5-day timescale.

To assess variability due to sample heterogeneity, five separate aliquots sampled from the same batch of freeze-dried kale microgreens were independently extracted and analysed on the same day to minimize instrumental drift. The resulting RSDs of normalized EIC peak areas reflected sampling-related variability within a homogenized lot obtained from approximately 600 seedlings (including leaves and stems). Although this variability inherently includes a minor contribution from the extraction procedure, recovery experiments indicated that this contribution was marginal relative to sampling variability. Consequently, data shown in the second column of Table S2 can be considered mainly as an estimate of the sampling-related variability, in turn associated with the level of homogeneity of the freeze-dried material obtained for each type of vegetal product under study. As was apparent, the resulting RSD was generally lower than 15%, being close to 20-21% only for two sterols among those considered.

Section S3. Details on the cultivation of kale and broccoli raab microgreens, microleaves and baby leaves

Plants were grown in plastic trays (microgreens) or pots (microleaves and baby leaves) filled with a peat-based commercial growing media (Brill® 3 Special). The seeds were uniformly broadcasted on the surface of the growing media using a seeding density of 4 seeds/cm^2 for microgreens, 0.076 seeds/cm^2 for microleaves and 0.038 seeds/cm^2 for baby leaves. During the first 4 days, trays and pots were covered with plastic film for seed germination. On day five, the seedlings were exposed to light inside the greenhouse. Trays and pots were irrigated every day using rainwater until the germination was complete. In both cycles, after germination, trays and pots were fertigated with a half-strength Hoagland nutrient solution with two interventions per day. In the case of microgreens, plants were harvested at the first true-leaf stage. In the first cultivation cycle, broccoli raab and kale microgreens were harvested 18 and 25 days after sowing, respectively; in the second cycle they were harvested 16 and 18 days after sowing, respectively. For microleaves, plants were harvested at the 4–5 true leaf stage; in the first cycle broccoli raab and kale were harvested 36 and 44 days after sowing, respectively; in the second cycle harvests occurred at 29 and 30 days after sowing, respectively. Baby leaves were grown only during the winter cycle and harvested at the 7-8 true leaf stage, corresponding to 49 and 58 days after sowing for broccoli raab and kale, respectively.

Section S4. Details on the cultivation and cooking of mature forms of kale and broccoli raab

Kale and broccoli raab plants were cultivated until they reached the mature form using first an N-based fertilizer, to promote the development and strengthening, and then with a K-based fertilizer, to promote fruiting, while the irrigation was carried out every other day with about 10 L of water. After harvest, plants were washed with tap water, blotted dry with paper towels and cut to obtain edible portions. In particular, plants for each genotype were mixed, with a total of 1350 g being taken and subsequently divided into three 450 g portions. From each of the latter, 150 g aliquots were obtained, one of which was retained raw, while each of the remaining two were cooked in a different way. In the first case (boiling) vegetables were boiled in a steel pot with distilled water (99 ± 1 ◦C) at a sample weight/water volume ratio of 1:7 for 5 min. The boiled samples were drained off and rapidly cooled on ice. In the second case (steaming) vegetables were placed on a tray in a steam cooker (VC 101 630, Tefal, Milan, Italy), covered with a lid and cooked with water vapour (99 ± 1 ◦C) under atmospheric pressure for 10 min. Cooking conditions were determined with a preliminary experiment. Based on the described experimental design, three independent replicates for each cooking method and as many for raw vegetables were obtained.

Tables

Table S1. Summary of data concerning the external calibration of standard sterols: Concentration range, calibration line equation and coefficient of determination (R^2). The LOD and LOQ values (µg/mL) were determined as 3 and 10 times the ratio of the calibration intercept standard deviation to the slope, respectively.

Compound	Explored range (µg/mL)	Calibration line	R^2	LOD (µg/mL)	LOQ (µg/mL)
β-sitosterol	0.1-100	y = 0.0466x + 0.0321	0.9991	2	7
Campesterol	0.1-50	y = 0.1378x - 0.0757	0.9992	1.2	4
Brassicasterol	0.01-10	y = 0.0564x + 0.0021	0.9997	0.11	0.4
Chalinasterol	0.01-10	y = 0.0855x - 0.0031	0.9999	0.08	0.3
Isofucosterol	0.01-10	y = 0.1272x + 0.0082	0.9995	0.15	0.5
Δ^7-avenasterol	0.01-10	y = 0.0577x + 0.0015	0.9999	0.08	0.3
Stigmasterol	0.001-5	y = 0.4153x + 0.0067	0.9994	0.04	0.14
Cholesterol	0.001-5	y = 0.0972x + 0.0041	0.9994	0.08	0.3
Lathosterol	0.001-5	y = 0.0901x - 0.0002	0.9999	0.03	0.11

Table S2. Percent relative standard deviation (RSD) values related to the sampling-extraction variability and the analytical response variability evaluated for standard sterols and for 24-epibrassicasterol in kale microgreens. Sampling-extraction variability was estimated from the extraction and LC-MS analysis of five separate aliquots sampled from the same batch of lyophilized material (corresponding to a specific sub-section of the greenhouse in which kale microgreens were cultivated) processed on the same day. The analytical response variability was assessed by repeating the LC-MS analysis of a single extract three times on alternate days within a week, with the extract being stored at −18°C between the analyses.

**Compound**	**Sampling-extraction variability RSD %**	**Analytical response variability RSD %**
β-sitosterol	15	4
Campesterol	12	0.8
Isofucosterol	15	4
Brassicasterol	20	3
Cholesterol	14	1.2
Chalinasterol	13	5
24-epibrassicasterol	21	4
Δ^7-avenasterol	15	6

Table S3. Free sterol contents (mg/100 g FW) of mature kale and broccoli raab after steaming or boiling in water. Values represent 95% confidence intervals centred on means (*n* = 3).

Plant	Cooking method	β-sitosterol	Campesterol	Isofucosterol	Cholesterol	Chalinasterol	Δ^7-avenasterol
Kale	Steamed	23 ± 6	2.6 ± 0.6	0.18 ± 0.08	< LOD	< LOD	0.18 ± 0.10
	Boiled	18 ± 7	1.8 ± 0.6	0.16 ± 0.06	< LOD	< LOD	0.11 ± 0.06
Broccoli raab	Steamed	22 ± 5	2.3 ± 0.8	0.42 ± 0.14	0.67 ± 0.17	1.1 ± 0.2	0.12 ± 0.03
	Boiled	27 ± 9	2.7 ± 1.3	0.9 ± 0.6	1.0 ± 0.4	3 ± 2	0.25 ± 0.17

Figures

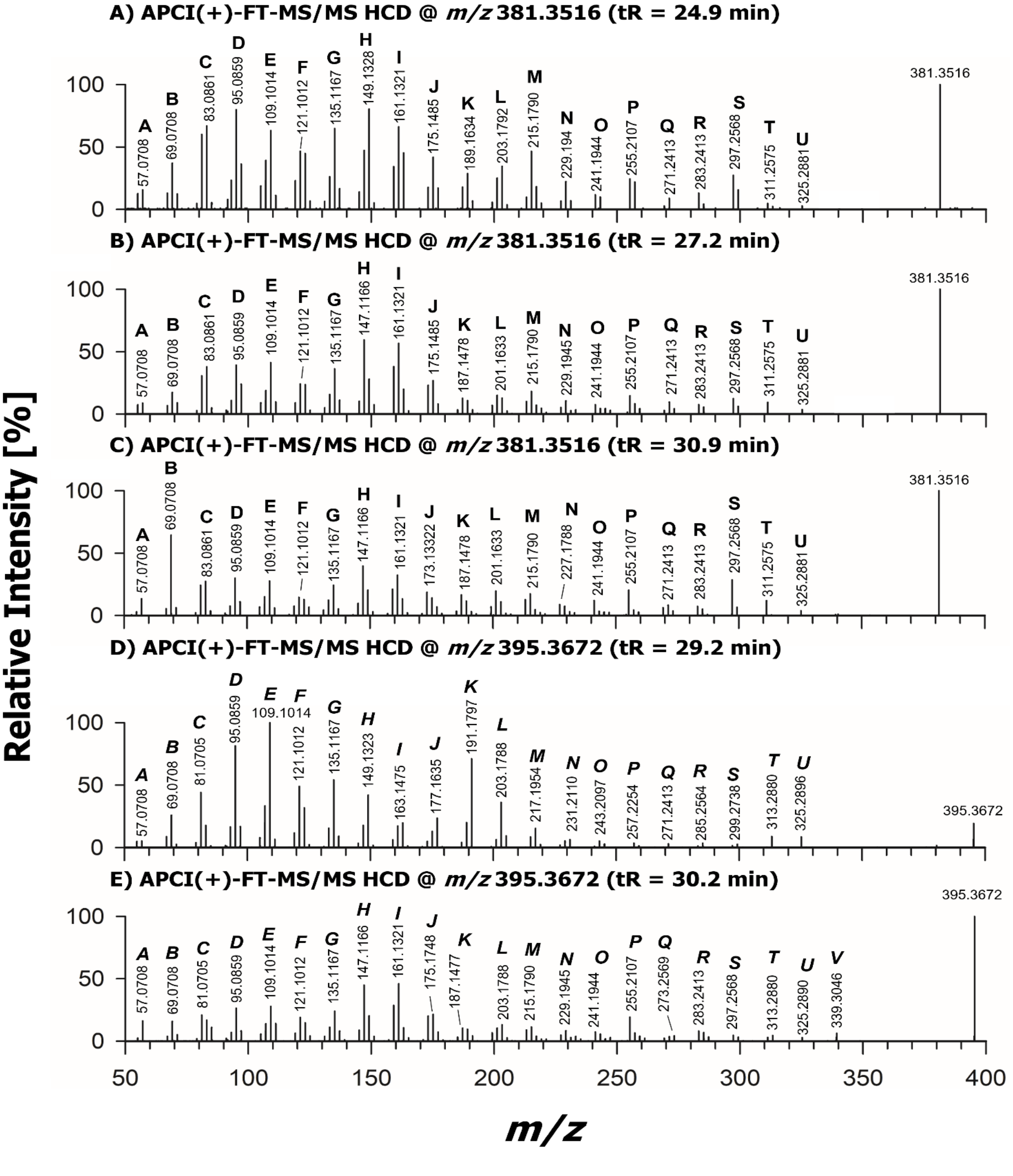

Figure S1. APCI(+)-HCD-HRMS/MS spectra acquired for the $[M + H - H_2O]^+$ ions of sterols whose peaks were detected at specific retention times in the EIC traces referred to exact *m/z* values 381.3516 (plots (A–C)) and 395.3672 (plots (D,E)) upon RPLC-APCI-HRMS analysis of a kale microgreens extract (see Figure 2). Peak signals of product ions sharing the same number of carbon atoms but differing in hydrogen atom count were grouped into clusters, each identified by a capital letter. The experimental *m/z* value of the most intense signal within each cluster is reported.

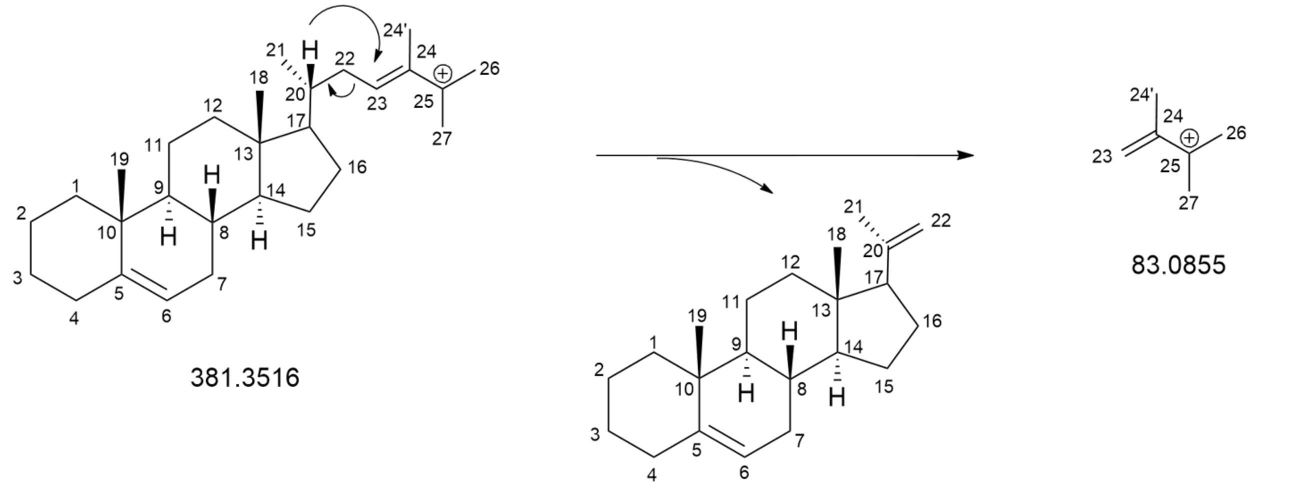

Figure S2. Proposed fragmentation mechanism for the generation of a product ion with exact *m/z* 83.0855, consistent with one of the peak signals detected in the APCI(+)-HCD-HRMS/MS spectrum of the sterol eluting at 27.2 min in the EIC chromatogram obtained for *m/z* 381.3516 upon RPLC-APCI(+)-HRMS analysis of a kale microgreen extract (Figure 2C). The sterol was tentatively identified as 24-methylcholesta-5,23E-dien-3β-ol, based on its MS/MS profile (see main text for details). Exact monoisotopic *m/z* ratios, rounded off to the fourth decimal place, are reported.

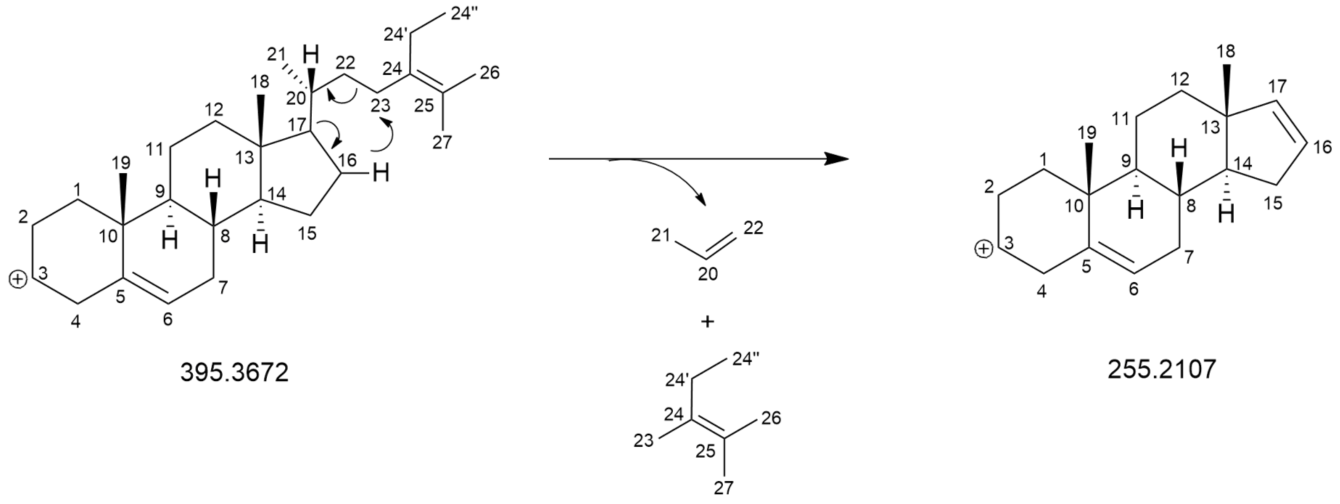

Figure S3. Proposed fragmentation mechanism for the generation of a product ion with exact *m/z* 255.2107, consistent with one of the peak signals detected in the APCI(+)-HCD-HRMS/MS spectrum of the sterol eluting at 30.2 min in the EIC chromatogram obtained for *m/z* 395.3672 upon RPLC-APCI(+)-HRMS analysis of a kale microgreen extract (Figure 2D). The sterol was tentatively identified as $\Delta^{5,24}$-stigmastadienol, based on its MS/MS spectrum. Exact monoisotopic *m/z* ratios, rounded off to the fourth decimal place, are reported.

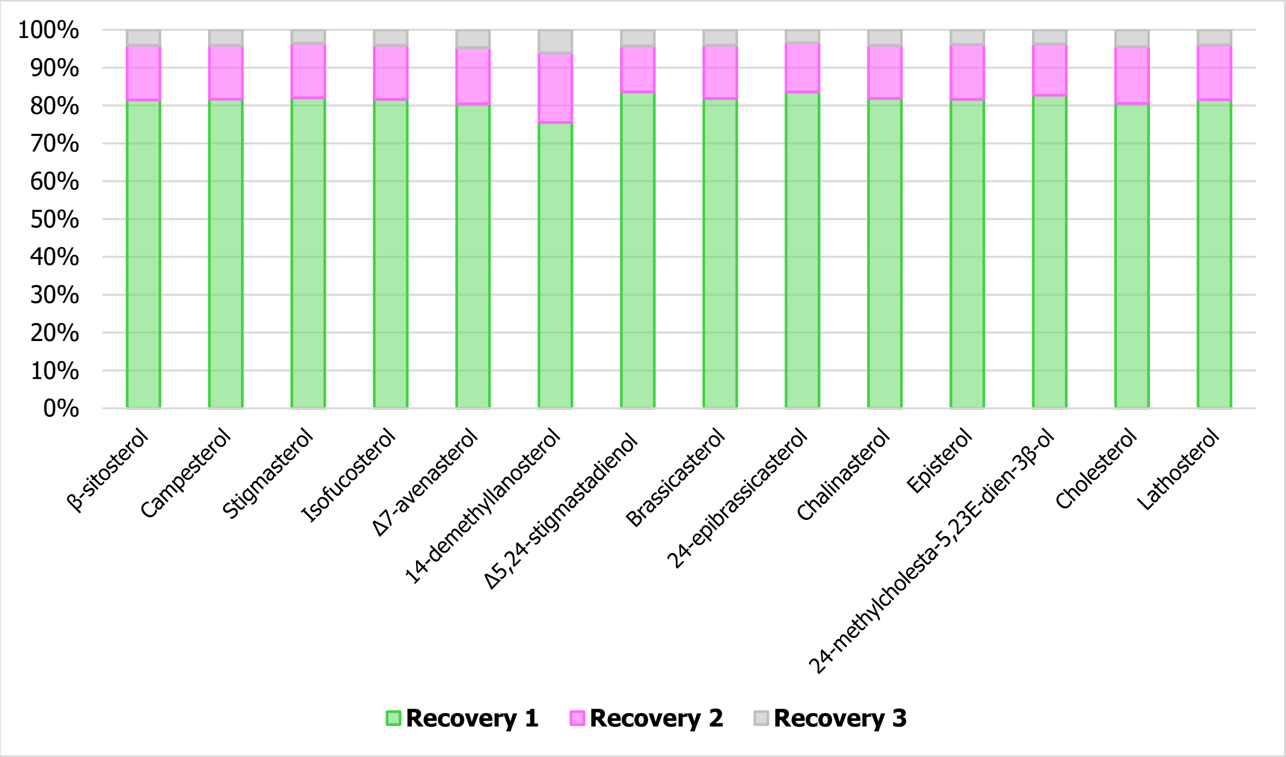

Figure S4: Stacked-bar graph reporting the average cumulative percentual recovery (n = 3) of main sterols detected in kale microgreens evaluated after each of three successive extraction steps.

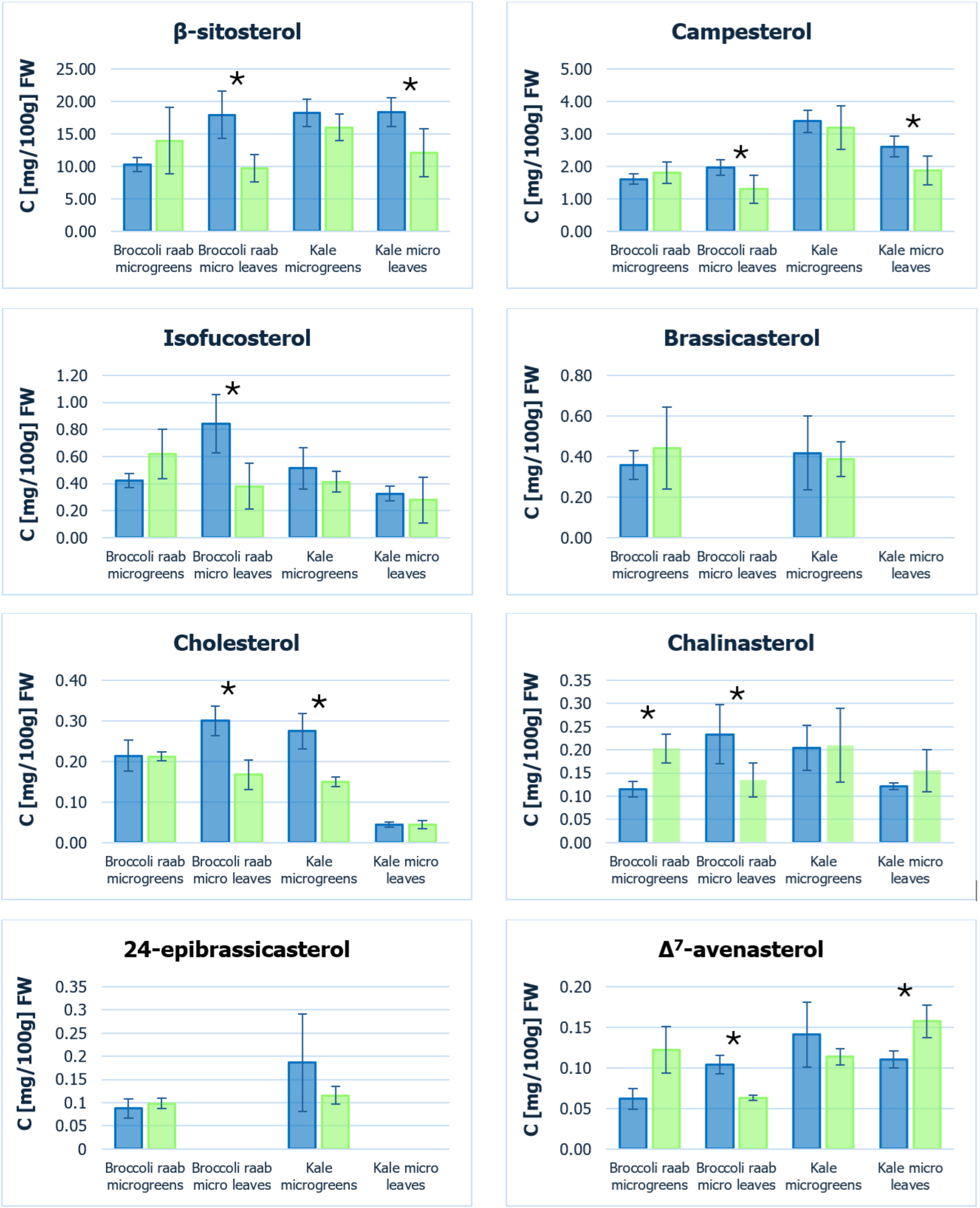

Figure S5. Column graphs showing average free sterol concentrations (mg/100 g FW) found in microgreens and microleaves of kale and broccoli raab grown in winter (blue bars) and spring (green bars). Error bars represent 95% confidence intervals ($n = 3$ for each product type). Asterisks indicate statistically significant differences between the two seasons ($p < 0.05$).

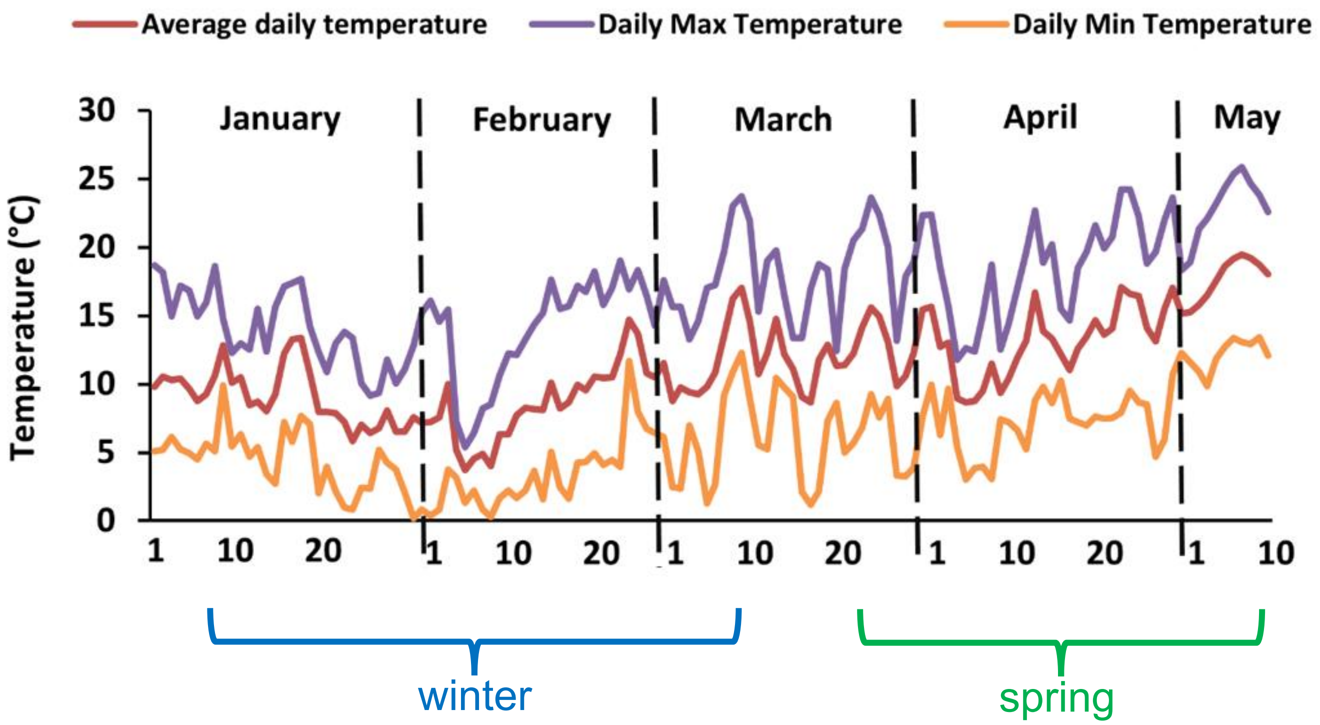

Figure S6. Average, minimum and maximum temperatures recorded inside the greenhouse during the two growing cycles (winter: 9 January–9 March 2023; spring: 21 March–9 May 2023) adopted for the cultivation of microgreens and microleaves of kale and broccoli raab.
